# Double-stranded RNA sequencing reveals distinct riboviruses associated with thermoacidophilic bacteria from hot springs in Japan

**DOI:** 10.1038/s41564-023-01579-5

**Published:** 2024-01-17

**Authors:** Syun-ichi Urayama, Akihito Fukudome, Miho Hirai, Tomoyo Okumura, Yosuke Nishimura, Yoshihiro Takaki, Norio Kurosawa, Eugene V. Koonin, Mart Krupovic, Takuro Nunoura

**Affiliations:** 1 Department of Life and Environmental Sciences, Laboratory of Fungal Interaction and Molecular Biology (donated by IFO), University of Tsukuba, Tsukuba, Japan; 2 Microbiology Research Center for Sustainability (MiCS), University of Tsukuba, Tsukuba, Japan; 3 Howard Hughes Medical Institute, Department of Biology and Department of Molecular and Cellular Biochemistry, Indiana University, Bloomington, IN USA; 4 Super-cutting-edge Grand and Advanced Research (SUGAR) Program, Japan Agency for Marine Science and Technology (JAMSTEC), Yokosuka, Japan; 5 Marine Core Research Institute, Kochi University, Nankoku, Kochi, Japan; 6 Research Center for Bioscience and Nanoscience (CeBN), JAMSTEC, Yokosuka, Japan; 7 Faculty of Science and Engineering, Soka University, Hachioji, Japan; 8 National Center for Biotechnology Information, National Library of Medicine, National Institutes of Health, Bethesda, MD USA; 9 Institut Pasteur, Université Paris Cité, Archaeal Virology Unit, Paris, France

**Keywords:** Virology, Microbial ecology

## Abstract

Metatranscriptome sequencing expanded the known diversity of the bacterial RNA virome, suggesting that additional riboviruses infecting bacterial hosts remain to be discovered. Here we employed double-stranded RNA sequencing to recover complete genome sequences of two ribovirus groups from acidic hot springs in Japan. One group, denoted hot spring riboviruses (HsRV), consists of viruses with distinct RNA-directed RNA polymerases (RdRPs) that seem to be intermediates between typical ribovirus RdRPs and viral reverse transcriptases. This group forms a distinct phylum, *Artimaviricota*, or even kingdom within the realm *Riboviria*. We identified viruses encoding HsRV-like RdRPs in marine water, river sediments and salt marshes, indicating that this group is widespread beyond extreme ecosystems. The second group, denoted hot spring partiti-like viruses (HsPV), forms a distinct branch within the family *Partitiviridae*. The genome architectures of HsRV and HsPV and their identification in bacteria-dominated habitats suggest that these viruses infect thermoacidophilic bacteria.

## Abstract (web summary)

Prokaryotic RNA viruses recovered from hot springs in Japan using fragmented and primer-ligated dsRNA sequencing form divergent clades in the *Riboviria* branch of RNA viruses.

## Main

Recent metagenomics and metatranscriptomics analyses transformed the study of viromes. These approaches that do not require laborious virus cultivation have become the principal source of virus discovery^1^. Indeed, numerous virus groups across all taxonomic levels have been discovered. In particular, the diversity of RNA viruses that, in the current virus taxonomy, comprise the kingdom *Orthornavirae* within the realm *Riboviria* has expanded more than an order of magnitude through global metatranscriptome surveys^2–9^.

Only one hallmark gene encoding the RNA-directed RNA polymerase (RdRP) is conserved across the entire kingdom *Orthornavirae*. Therefore, detection of the RdRP, typically using search methods based on sequence profiles, is the principal approach employed in metatranscriptome mining for riboviruses, and phylogenetic analysis of the RdRP is the basis of ribovirus taxonomy. Before the advent of massive metatranscriptome analysis, the viruses in this kingdom have been classified into 5 large phyla corresponding to major clades in the RdRP phylogeny^10^. Metatranscriptome studies largely validated the robustness of these phyla and additionally identified several candidate smaller phyla. The diversity of riboviruses across the lower taxonomy ranks demonstrated a nearly uniform increase, for example, roughly fivefold in one study that provided quantitative estimates^8^.

Metatranscriptome mining yielded qualitative insights into the global view of the RNA virome. Traditionally, riboviruses have been recognized as the major component of the eukaryote virome, whereas the viromes of bacteria and archaea were dominated by DNA viruses^11,12^. For many years, only two small families of RNA viruses, each infecting a narrow range of bacteria, have been known: *Leviviridae* (single-stranded RNA (ssRNA) bacteriophages) and *Cystoviridae* (double-stranded RNA (dsRNA) bacteriophages). Metatranscriptome analyses revealed a much greater diversity of leviviruses than previously suspected, elevating this family to the rank of the class *Leviviricetes* that includes multiple orders and families^8,13–15^. The family *Cystoviridae* was substantially expanded as well^8^. For uncharacterized groups of viruses without a close relationship to any known groups, host assignment becomes a challenge. Nevertheless, several lines of evidence including (nearly) exclusive co-occurrence with bacteria, prediction of multiple virus genes preceded by prokaryote-type (Shine–Dalgarno (SD)) ribosome-binding sequences (RBS), identification of virus-encoded cell wall degrading enzymes, and most notably, targeting by reverse transcriptase (RT)-containing type III CRISPR systems strongly suggest that several previously uncharacterized groups of riboviruses infect prokaryotes^8^. Thus, the diversity of riboviruses infecting bacteria has been substantially underestimated and additional groups of such viruses most probably remain to be discovered.

Long dsRNA is a molecular marker of RNA virus infection^16^. The recently developed method of Fragmented and primer-Ligated DsRNA Sequencing (FLDS) made it possible to capitalize on the presence of (nearly) identical terminal sequences in genome segments of the same virus. This information enables one to identify multisegmented RNA virus genomes even if they did not show sequence similarity to known viruses^17–19^. Here we used FLDS to identify riboviruses associated with microbial consortia dominated by bacteria and archaea in several acidic hot springs in Japan. This analysis resulted in the identification of two distinct groups of riboviruses with multisegmented RNA genomes with organization typical of bacterial riboviruses.

## Composition of small subunit ribosomal RNA and identification of RNA virus

**Fig. 1:**
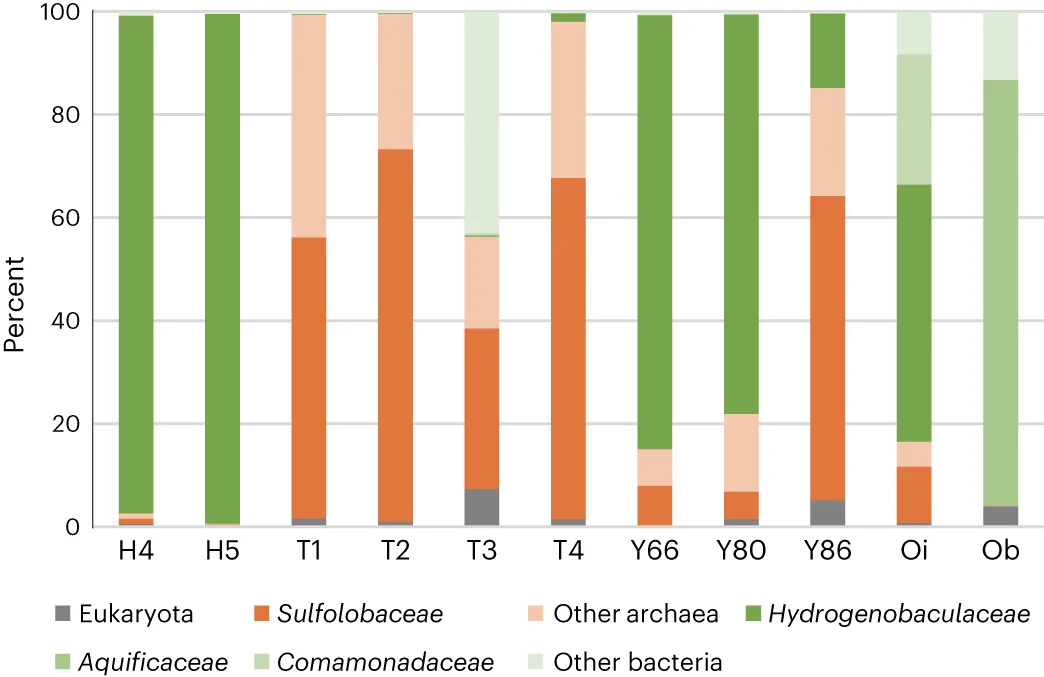
Composition of microbiomes associated with the hot spring samples. The composition was analysed on the basis of the mapped sequence reads on the rRNA sequences using Phyloflash. Details are given in Extended Data Table 1.

To determine the composition of active microbial consortia in the hot spring water samples, total ssRNA sequencing reads were mapped on the small subunit (SSU) ribosomal RNA (rRNA) sequences from the Silva database (SILVA SSU v.138) using phyloFlash^20^ (Fig. 1 and Supplementary Text). All samples were dominated by prokaryotes, with the H4, H5, Y66 and Oi samples, where RNA viruses were identified, containing <1% of eukaryotic SSU rRNA reads (Extended Data Table 1).

In FLDS, potential complete genomes of multipartite RNA viruses were obtained from samples H4, H5, Y66 and Oi (Extended Data Table 2). For the samples from the other stations, sequence libraries were successfully constructed except for the Ob sample, but no contigs representing potential complete genomes of RNA viruses in FLDS read mapping^18^ were obtained.

## Bipartite RNA virus from the hot spring and other ecosystems

**Fig. 2:**
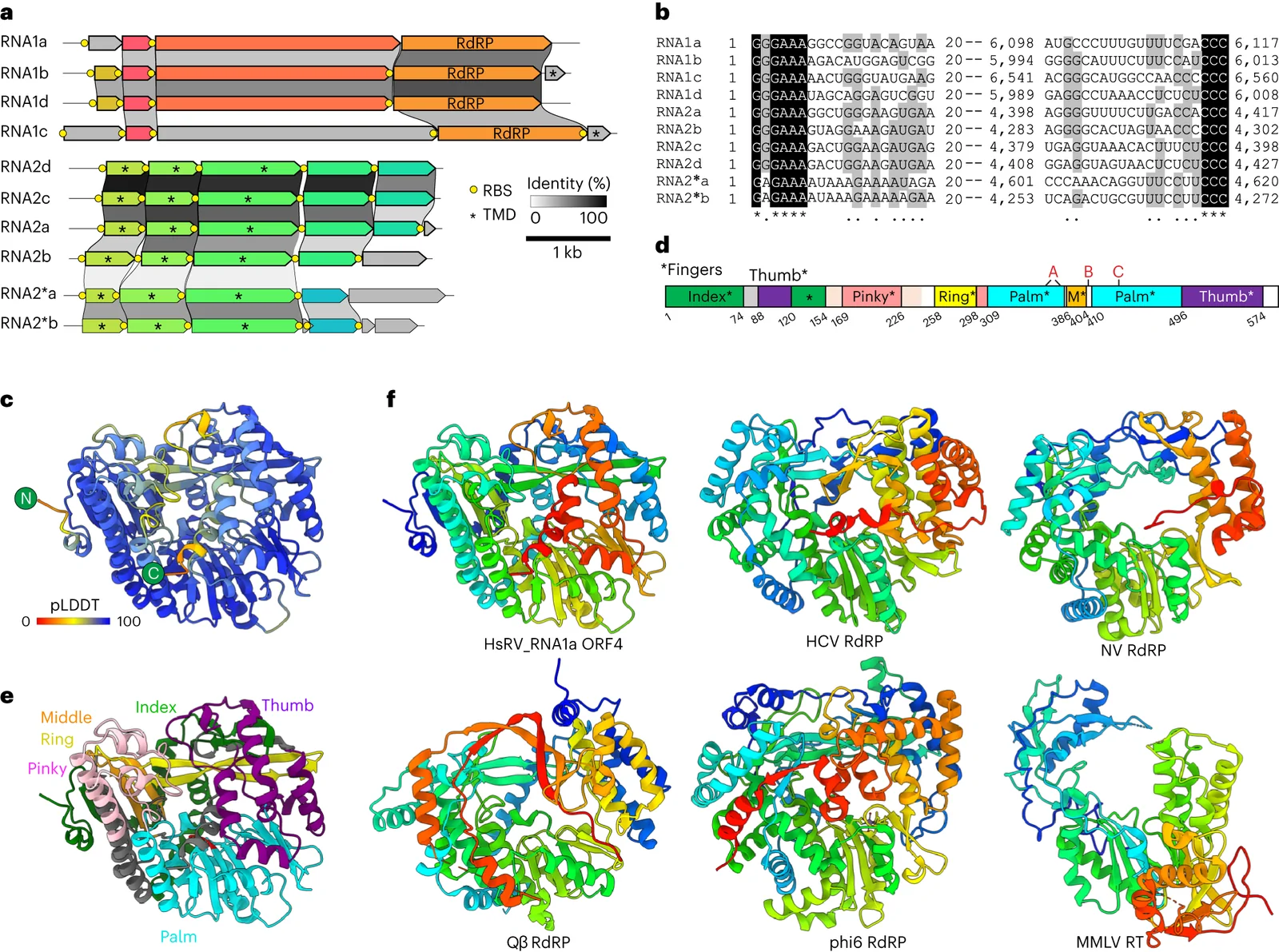
Unusual bipartite RNA virus genomes from the Oi hot spring. **a**, Genome organization and conservation of the three genomic segments (RNA1, RNA2 and RNA2*) of HsRV. ORFs encoding homologous proteins are shown as arrows with identical colours. Yellow circles represent predicted SD RBS. Asterisks denote putative genes encoding predicted transmembrane domain (TMD)-containing proteins. **b**, MSA of the 5ʹ- and 3ʹ-terminal regions of the coding strands of reconstructed genome segments. Black shading, 100% nucleotide identity; grey shading, >50% nucleotide identity. **c**, Quality assessment of the AlphaFold2 model of the HsRV RdRP. The structural model is coloured on the basis of the pLDDT scores (average pLDDT = 90.7), with the colour key shown at the bottom left corner. **d**,**e**, Domain organization of the HsRV RdRP. **d**, Schematic representation of the domain organization, with exact coordinates of each subdomain, including the five ‘Fingers’, indicated. M, middle finger. The positions of the motifs A, B and C are indicated. **e**, The structural model of HsRV RdRP coloured using the same scheme as in **d**. **f**, Comparison of the HsRV RdRP with homologues from other RNA viruses, including hepatitis C virus (HCV; PDB: 6GP9), Norwalk virus (NV; PDB: 1SH0), Qβ (PDB: 3MMP), phi6 (PDB: 1HHS) as well as RT from Moloney murine leukaemia virus (MMLV; PDB: 4MH8). The structures are coloured using the rainbow scheme, from blue N terminus to red C terminus.

FLDS of the Oi sample (79.3 °C, pH 2.2) yielded three populations of contigs (Fig. 2a) which collectively recruited ∼50% of the clean FLDS reads from the Oi library. Among the contigs, we identified similar 5′- and 3′-terminal sequences (Fig. 2b), a characteristic feature of segmented RNA viruses^21^. On the basis of the similarity of the 5′- and 3′-terminal sequences, lengths of the segments and gene content, we concluded that two sets of contigs constituted genomes of a distinct group of bipartite RNA viruses. The segments were denoted RNA1, RNA2 and RNA2* (Supplementary Text and Extended Data Table 3). In total, we obtained complete sequences for 4, 4 and 2 divergent variants of segments RNA1, RNA2 and RNA2*, respectively (Fig. 2a). The similarity between the termini of the segments precluded assignment of all sets of segments to particular virus strains. However, segments RNA1a and RNA2a were most abundant and had longer conserved terminal sequences and were thus assigned to the same virus strain with a bisegmented genome.

**Extended Data Fig. 1:**
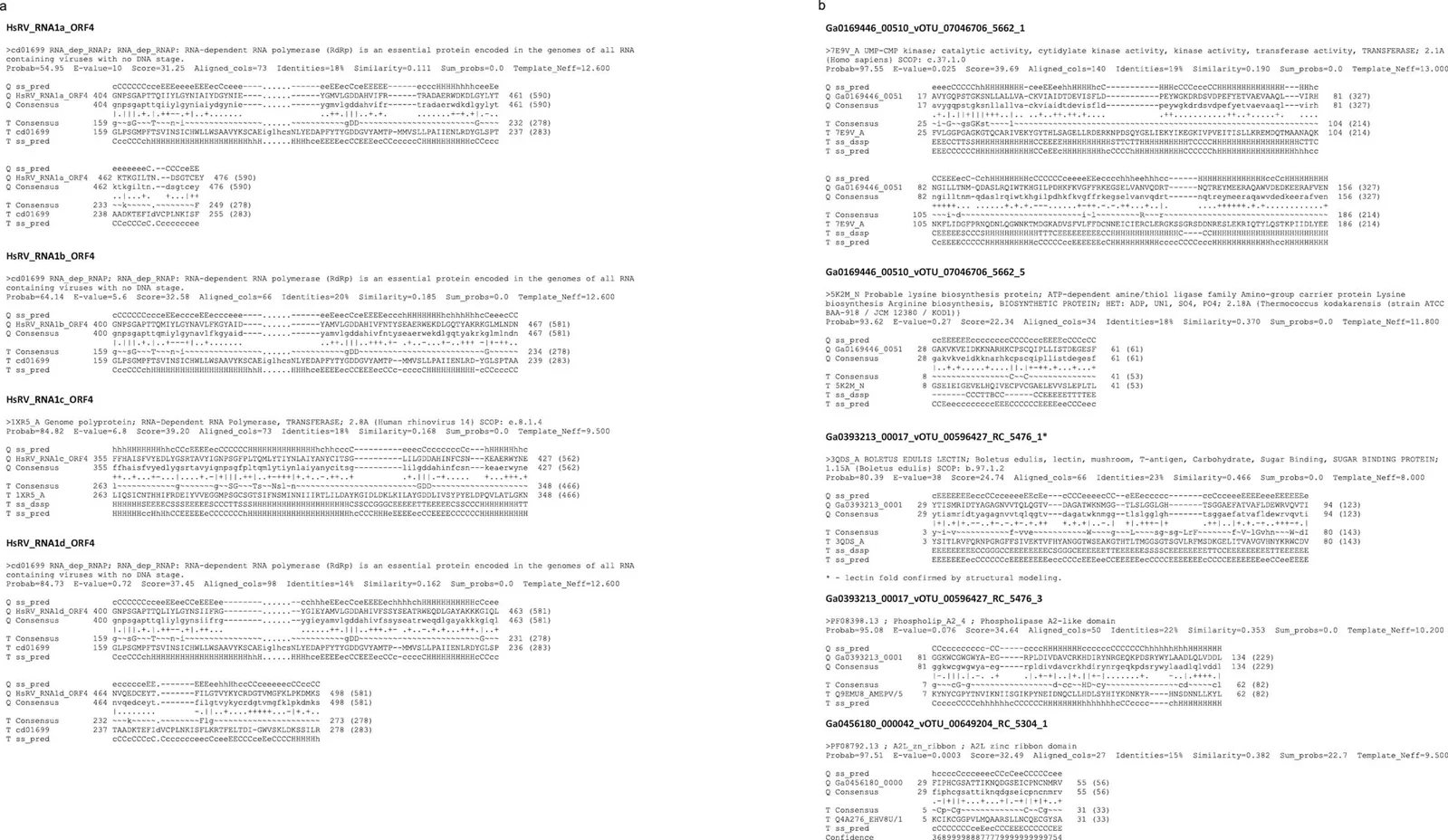
Results of the HHsearch analysis. Results of the HHsearch analysis queried with the **a**, putative ORF4 protein sequences from HsRV virus strains, and **b**, indicated protein sequences encoded by HsRV-like viruses from moderate ecosystems. H(h), α-helix; E(e), β-strand; C(c), coil.

RNA1, RNA2 and RNA2* harboured 4–5, 5–6 and 5–7 open reading frames (ORFs), respectively (Fig. 2a). None of the predicted proteins encoded by these RNAs showed significant similarity (BLASTP *E*-value = 5 × 10^−03^) to any protein sequences in public databases. Even the most sensitive profile–profile searches using HHpred yielded no significant (HHpred probability >90%) hits for any of the predicted proteins. However, HHpred searches queried with the amino acid sequence of ORF4 from the RNA1 segment produced a partial hit to several RdRPs. Although the hits were not significant (HHpred probability <90%) and encompassed only a small region of the RdRP (∼15% of the target profile), the aligned region covered the diagnostic RdRP motifs B (SGxxxT, x – any amino acid) and C (GDD) (Extended Data Fig. 1a), so we pursued this clue further. However, despite several attempts, we were unable to convincingly identify RNA1_ORF4 of HsRV as an RdRP (Supplementary Text). Thus, we set out to enrich the sequence diversity of RNA1_ORF4 by reanalyzing the entire FLDS dataset. To this end, unmapped sequence reads were assembled and RNA1_ORF4 protein sequences were used as queries to search against the assembled contigs using BLASTX. This search yielded 10 additional RNA1_ORF4-like sequences encoded by H5_contig_1 from H5 and Oi_contigs_1–9 from Oi samples (*E*-value ≤ 1 × 10^−05^) (Extended Data Table 4). The additional homologues detected in this search were combined with the 4 initially identified RNA1_ORF4 sequences and the produced multiple sequence alignment (MSA) was used as a query in an HHpred search against the PDB70 database. This search yielded significant hits (probability >90%) to various ribovirus RdRPs, although the aligned region remained limited (∼15% of the target profiles). Collectively, these searches suggested that RNA1_ORF4 homologues are highly divergent RdRPs.

**Extended Data Fig. 2:**
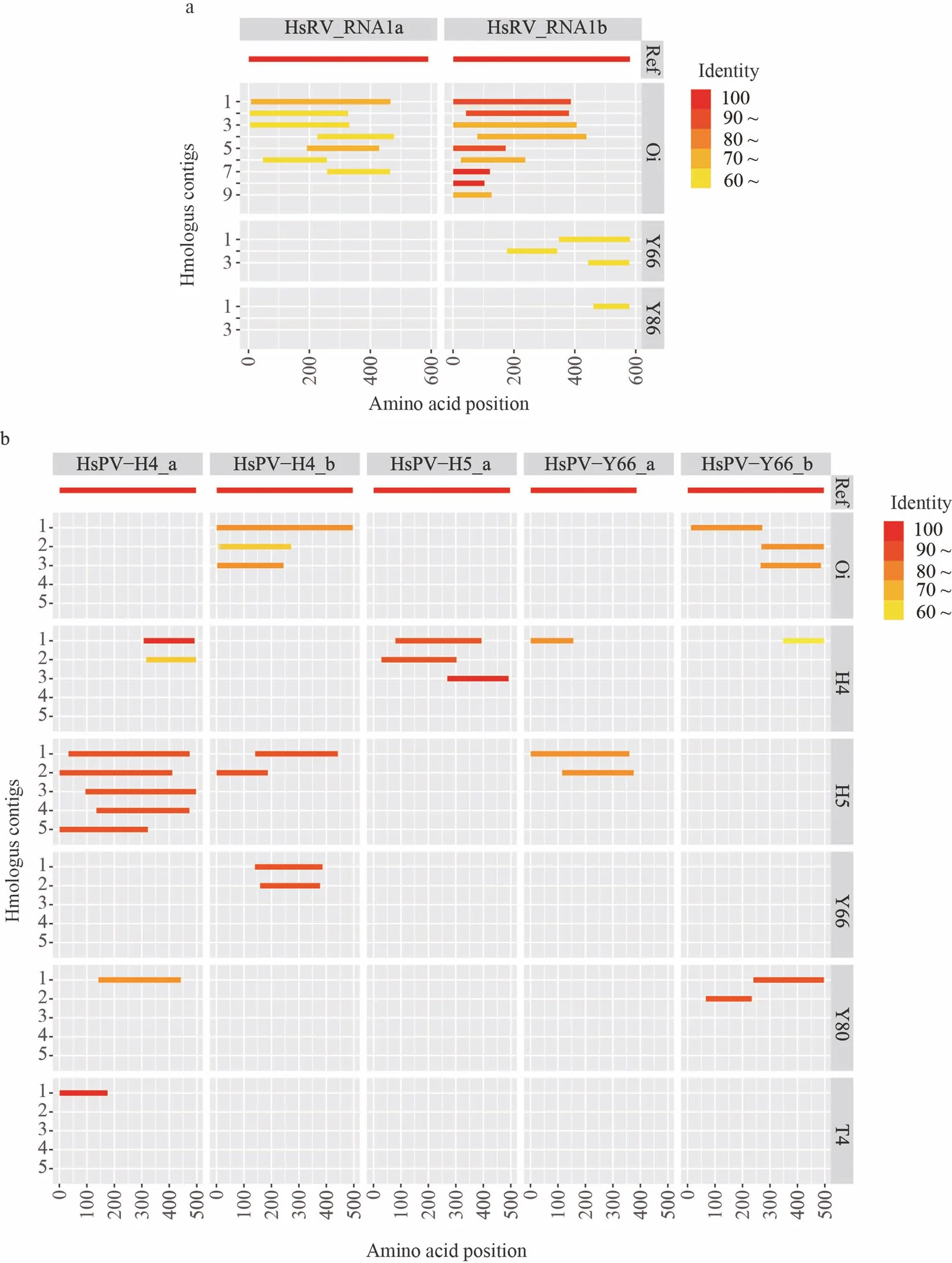
Distribution of minor contigs related to the RdRPs of HsRV and HsPV. Distribution of minor contigs related to the RdRPs of a, HsRV and b, HsPV. Each bar represents the position of predicted amino acid sequences of contigs aligned to the HsRV or HsPV RdRP shown at the top of the panel, and their identities to the reference RdRP sequences are indicated by the colors in the heatmap. The name of source libraries are shown in the right-side panel. Trimmed reads from each sample were assembled using CLC assembler, followed by the removal of sequences identical to HsRV or HsPV. Using the amino acid sequences of RdRPs from HsRV and HsPV as queries, tBLASTN searches were performed on the remaining contigs. Sequences with > 60% amino acid identity and > 100 aa hit were shown.

Using the MSA that included the identified RNA1_ORF4 homologues, a high-quality (average per-residue Local Distance Difference Test (pLDDT) = 90.7) AF2 model of the putative RdRP was obtained (Fig. 2c). Examination of this model revealed a topology typical of the palm-domain polymerases, with readily discernible ‘Fingers’, ‘Palm’ and ‘Thumb’ subdomains (Fig. 2d,e) and overall architecture similar to that of viral RdRPs (Fig. 2f), albeit with some unique structural features. In particular, the RNA1_ORF4 model displayed an extended and highly ordered ‘Fingers’ subdomain, with the ‘fingertips’ forming a 5-stranded β-sheet that is missing in other RdRPs and interacts with the ‘Thumb’ subdomain. The conserved motifs B and C identified by HHpred were located within the Palm subdomain, at positions equivalent to those in other RdRPs. Structural superposition of the Palm subdomains from different RdRPs allowed identification of the third core motif, A, in RNA1_ORF4 (see below). Thus, we concluded that RNA1_ORF4 encodes an RdRP and provisionally named the discovered bipartite virus ‘hot spring RNA virus (HsRV)’, with the strain harbouring segments RNA1a and RNA2a denoted HsRV1. The four RdRPs encoded by the complete RNA1 segments shared 37 to 75% pairwise amino acid sequence identity and thus appear to represent four distinct virus species (or even higher taxa). To characterize the diversity of HsRV-related RdRP in our FLDS data, the minor contigs including the aforementioned 10 sequences were analysed (Extended Data Fig. 2a). This analysis yielded several contigs with a high (>90%) identity to HsRV_RNA1b RdRP. In addition, several contigs with moderate (>60%) identity to HsRV_RNA1a or _RNA1b were detected. Y66 and Y86 also included a few contigs related to HsRV RdRP.

**Fig. 3:**
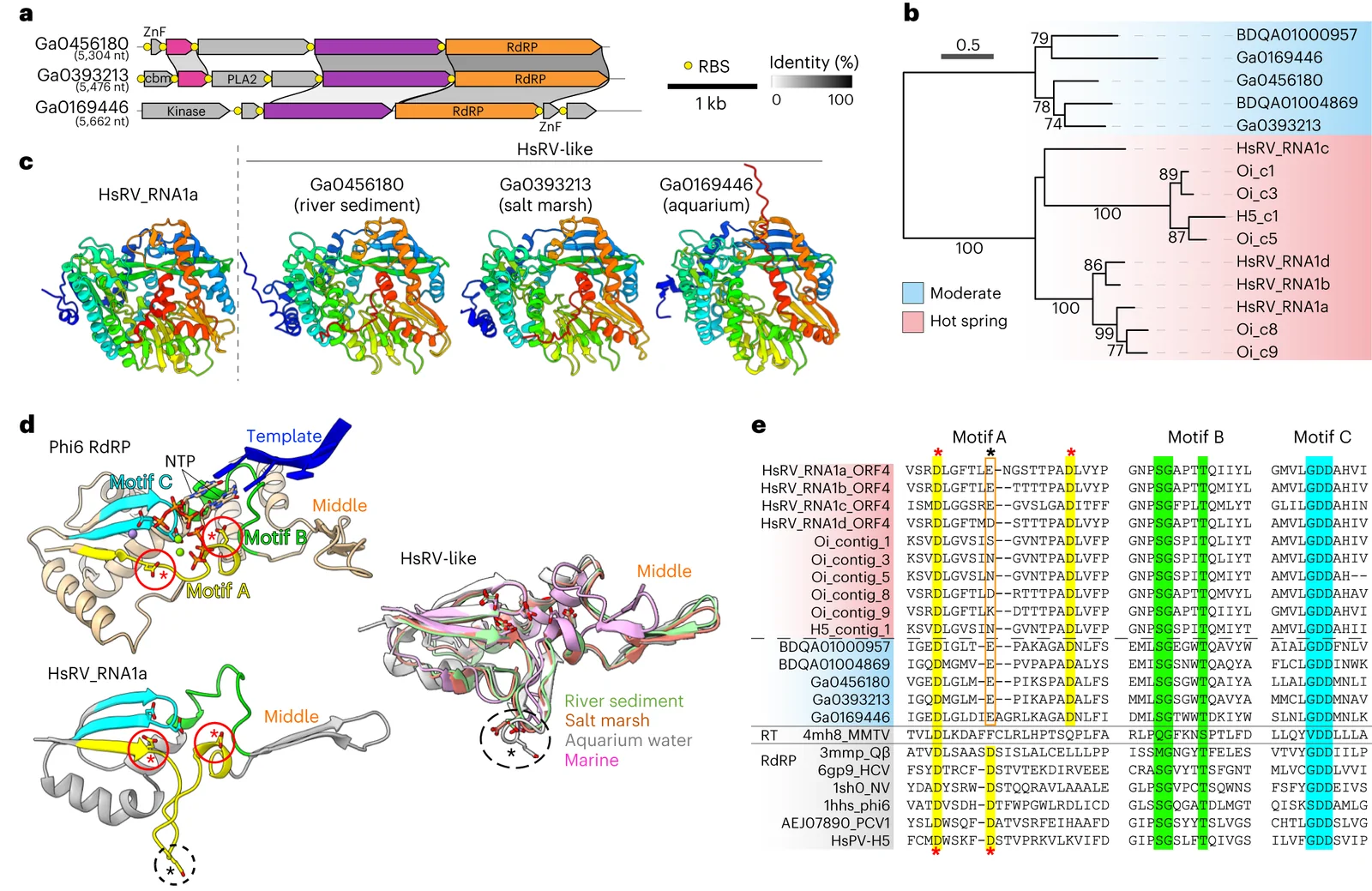
HsRV-like viruses from moderate environments. **a**, RdRP-encoding segments of HsRV-like viruses from non-extreme aquatic ecosystems. ORFs encoding homologous proteins are shown as arrows with identical colours. **b**, Maximum-likelihood phylogeny of the HsRV-like RdRPs encoded by viruses from extreme (pink) and moderate (blue) ecosystems. Node support was assessed using the SH-aLRT, with the corresponding values (%) shown on the branches. The scale bar represents the number of substitutions per site. **c**, Comparison of the HsRV RdRP with the homologues encoded by viruses from moderate aquatic ecosystems. The model was produced using AlphaFold2. The models are coloured using the rainbow scheme, from blue N terminus to red C terminus. **d**, Comparison of the catalytic cores encompassing the conserved RdRP motifs A (yellow), B (green) and C (cyan). Top: the structure of bacteriophage phi6 RdRP with the substrate nucleoside triphosphates (NTP) and template RNA strand (blue ribbon). Bottom: the HsRV RdRP. Middle: structurally superposed HsRV-like RdRPs from moderate ecosystems. The NTP and active site residues of motifs A and C are shown using the stick representation. The conserved aspartate residues of motif A are circled, with structurally equivalent residues indicated with red asterisks, whereas the non-conserved residue located in the loop facing away from the motif C in HsRV and related RdRP is indicated with the black asterisk. **e**, MSA of the conserved motifs of HsRV-like RdRPs from extreme (red shading) and moderate (blue shading) ecosystems with the corresponding regions from RdRPs and RT from other viruses (grey shading), including Moloney murine leukaemia virus (MMLV), hepatitis C virus (HCV), Norwalk virus (NV), PCV1 and hot spring partiti-like virus H5 (HsPV-H5). The sequences are indicated with the PDB or GenBank accession numbers. The conserved residues are shaded yellow, green and cyan, respectively, matching those in **d**. The conserved aspartate residues of motif A are highlighted in yellow, with structurally equivalent residues indicated with red asterisks, whereas the non-conserved residue in HsRV-like RdRPs located at the equivalent position as the second aspartate in other RdRPs is indicated with the black asterisk.

The sequence profile of the HsRV RdRP was used to search the previously described FLDS sequence data from coastal seawater samples^19^, leading to the identification of two additional contigs (GenBank accessions: BDQA01000957 and BDQA01004869) encoding incomplete HsRV-like RdRPs. Searches against the IMG/VR database queried with these RdRPs yielded significant hits (*E*-value ≤ 1 × 10^−05^) to three additional putative RdRPs encoded by apparently complete or near-complete 5.3–5.6-kb-long genome segments (Ga0456180_000042, Ga0393213_00017, Ga0169446_00510; Fig. 3a, Extended Data Fig. 1b, Table 5 and Supplementary Text).

Ga0456180, Ga0393213 and Ga0169446 originate from floodplain (river sediments), salt marsh and aquarium samples, respectively. Phylogenetic analysis of HsRV-like RdRPs showed clear separation between viruses from the hot spring and those from moderate aquatic environments (Fig. 3b). Collectively, these results indicate that HsRV-like viruses are broadly distributed in both hot springs and non-extreme aquatic ecosystems.

### Structural similarities between HsRV-like RdRPs and RTs

AF2 models of the three HsRV-like RdRPs from moderate ecosystems showed clear structural similarity with the HsRV RdRP, including the extended ‘Fingers’ subdomain (Fig. 3c). Another signature feature of these proteins is an unusual, extended RdRP motif A. In the canonical motif A, the two conserved Asp residues involved in catalysis and substrate discrimination^22,23^, respectively, are separated by 4–5 residues and bracket the catalytic GDD residues of motif C (Fig. 3d,e). By contrast, in HsRV-like RdRPs, the second Asp residue of motif A is not conserved, and the corresponding residue is located in a loop facing perpendicularly away from motif C, suggesting that it cannot perform the same function. However, all analysed HsRV-like RdRPs contain an Asp (Asp*) which is located 12–14 residues away from the first Asp of motif A (Fig. 3e). Despite the extended spacing in the protein sequence, Asp* occupies a position equivalent to that of the second Asp of the canonical motif A (Fig. 3d,e) and is likely to be its counterpart involved in substrate discrimination.

**Fig. 4:**
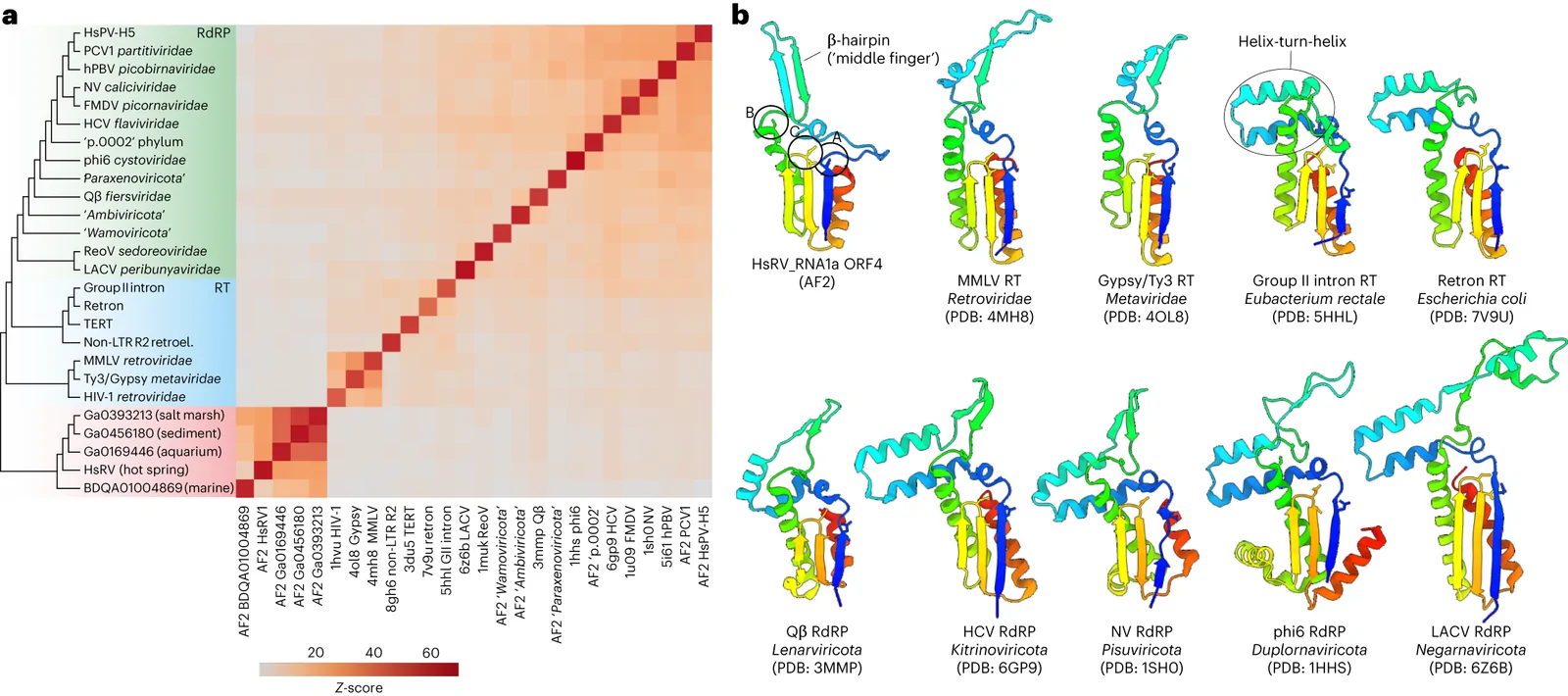
Structural relationships between RdRPs and RTs. **a**, Matrix and cluster dendrogram were constructed on the basis of the pairwise *Z*-score comparisons calculated using DALI. Different protein groups are highlighted with different background colours on the dendrogram: green, RdRPs from previously characterized viruses; blue, viral and non-viral RTs; red, HsRV-like RdRPs. The colour scale indicates the corresponding *Z*-scores. hPBV, human picobirnavirus; FMDV, foot-and-mouth disease virus; ReoV, reovirus; LACV, La Crosse virus; HIV-1, human immunodeficiency virus 1; TERT, telomerase RT; non-LTR R2 retroel., non-long terminal repeat R2 retroelement; AF2, AlphaFold2 model. For experimentally determined structures, the corresponding PDB accession numbers are indicated at the bottom of the matrix. **b**, Structural comparison of the core domain of RdRPs and RT encompassing the conserved motifs A–C. The structures are coloured using the rainbow scheme, from blue N terminus to red C terminus.

We next performed structural clustering on the basis of the pairwise DALI *Z*-scores of the HsRV-like RdRPs together with selected RdRPs of other riboviruses, including putative phyla of RNA phages identified in recent metatrascriptome analyses^7,8,24^ and RT encoded by eukaryotic viruses of the order *Ortervirales*^25^ as well as non-viral RTs from bacteria and eukaryotes (Fig. 4a). The HsRV-like RdRPs from both hot springs and moderate aquatic ecosystems formed a tight cluster, underscoring their relatedness despite high sequence divergence. All previously known viral RdRPs formed a clade in the structure-based dendrogram, but the HsRV-like RdRPs remained separated from those (Fig. 4a). The two viral RdRP clusters were interspersed with the RTs, such that the viral RTs were the closest structural neighbours of the HsRV-like RdRPs. This result confirms the extreme divergence of the HsRV-like RdRPs and might reflect a closer relationship to viral RTs. This unexpected link was strengthened by the comparison of the ‘Palm’ subdomain of HsRV-like RdRPs with homologues from other riboviruses as well as viral and non-viral RTs. In RdRPs of riboviruses from 5 established phyla^10^, the first β-strand (blue in Fig. 4b) containing motif A and the motif B-containing α-helix are separated by a characteristic helix-turn-helix (HTH) region followed by a β-hairpin corresponding to the ‘Middle’ finger subdomain (Fig. 2d,e). However, the HTH motif is absent in both the HsRV-like RdRPs and viral RTs. Notably, non-viral RTs, such as those from group II introns or retrons, contain the HTH motif but lack the β-hairpin region, which is compatible with the intermediate position of RTs between the two clades of viral RdRPs. Thus, the HsRV-like RdRPs might comprise an evolutionary intermediate between viral RdRPs and RTs. A BLASTN search against the metagenomic DNA sequences obtained from the hot springs did not detect HsRV-like sequences, suggesting that HsRV-like viruses are bona fide riboviruses that lack a DNA intermediate stage (Supplementary Text).

## A thermoacidophilic partiti-like virus

**Fig. 5:**
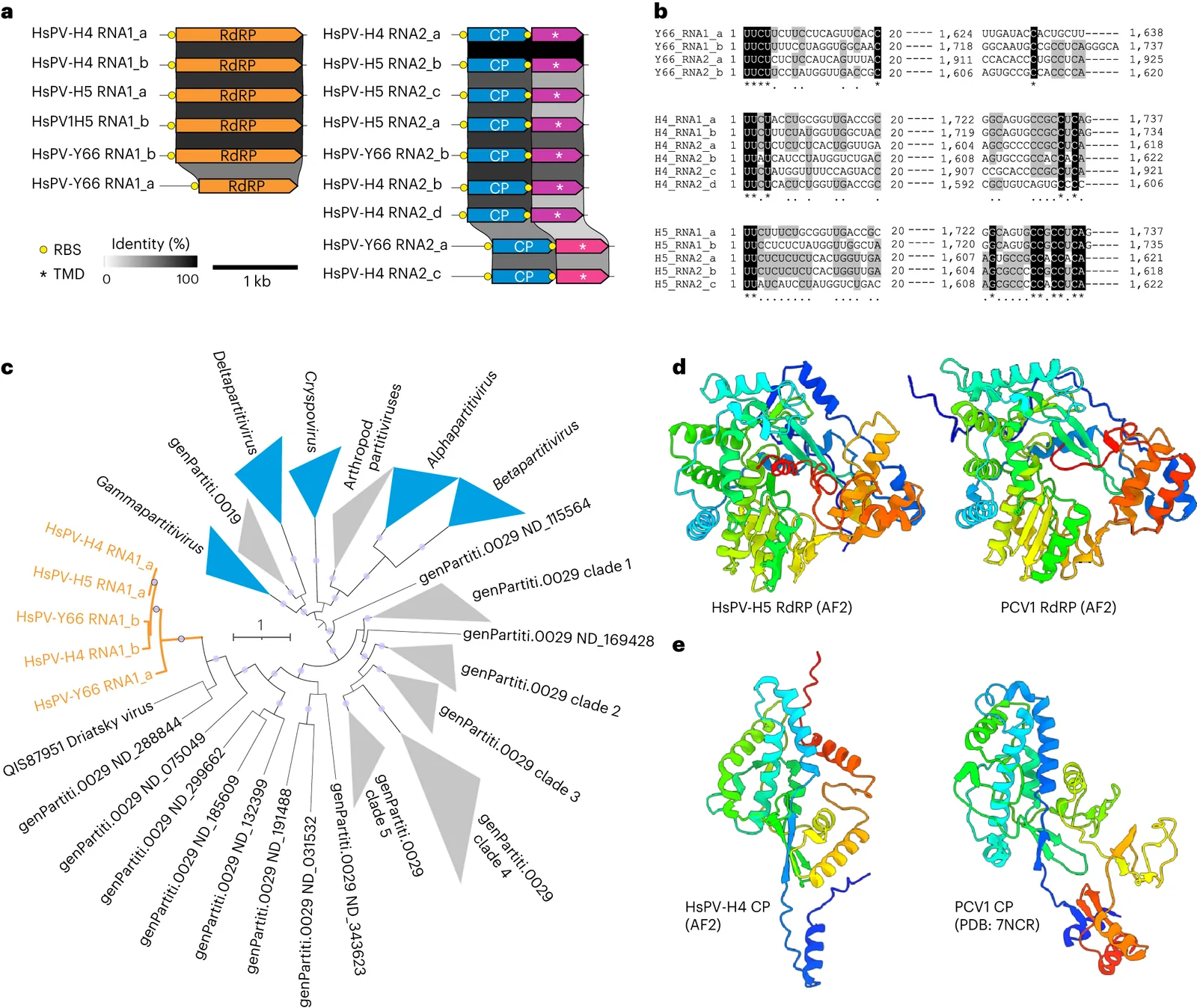
A thermoacidophilic partiti-like virus. **a**, Genome organization and conservation of the two genome segments, RNA1 and RNA2, of HsPV. ORFs encoding homologous proteins are shown as arrows with identical colours. Yellow circles represent predicted SD RBS. Asterisks denote putative genes encoding predicted TMD-containing proteins. **b**, MSA of the 5ʹ- and 3ʹ-terminal regions of the coding strands of reconstructed genome segments. Black shading, 100% nucleotide identity; grey shading, >50% nucleotide identity. **c**, Maximum-likelihood phylogeny of the RdRP proteins from representative members of the family *Partitiviridae* and related sequences (including all HsPV strains, shown in orange). Clades corresponding to the official *Partitiviridae* genera are shown in blue, whereas those corresponding to unclassified groups are in grey. Node supports were assessed using the SH-aLRT; circles indicate nodes with ≥90% supports. The scale bar represents the number of substitutions per site. GenBank accession numbers of used sequences are shown in Supplementary Text. **d**, Comparison of the RdRP from HsPV-H5 with a homologue from deltapartitivirus PCV1. **e**, Comparison of the CP from HsPV-H4 with a homologue from deltapartitivirus PCV1. The structures are coloured using the rainbow scheme, from blue N terminus to red C terminus. The HsPV RdRP and CP structures coloured on the basis of the pLDDT quality scores can be found in Extended Data Fig. 3.

**Extended Data Fig. 3:**
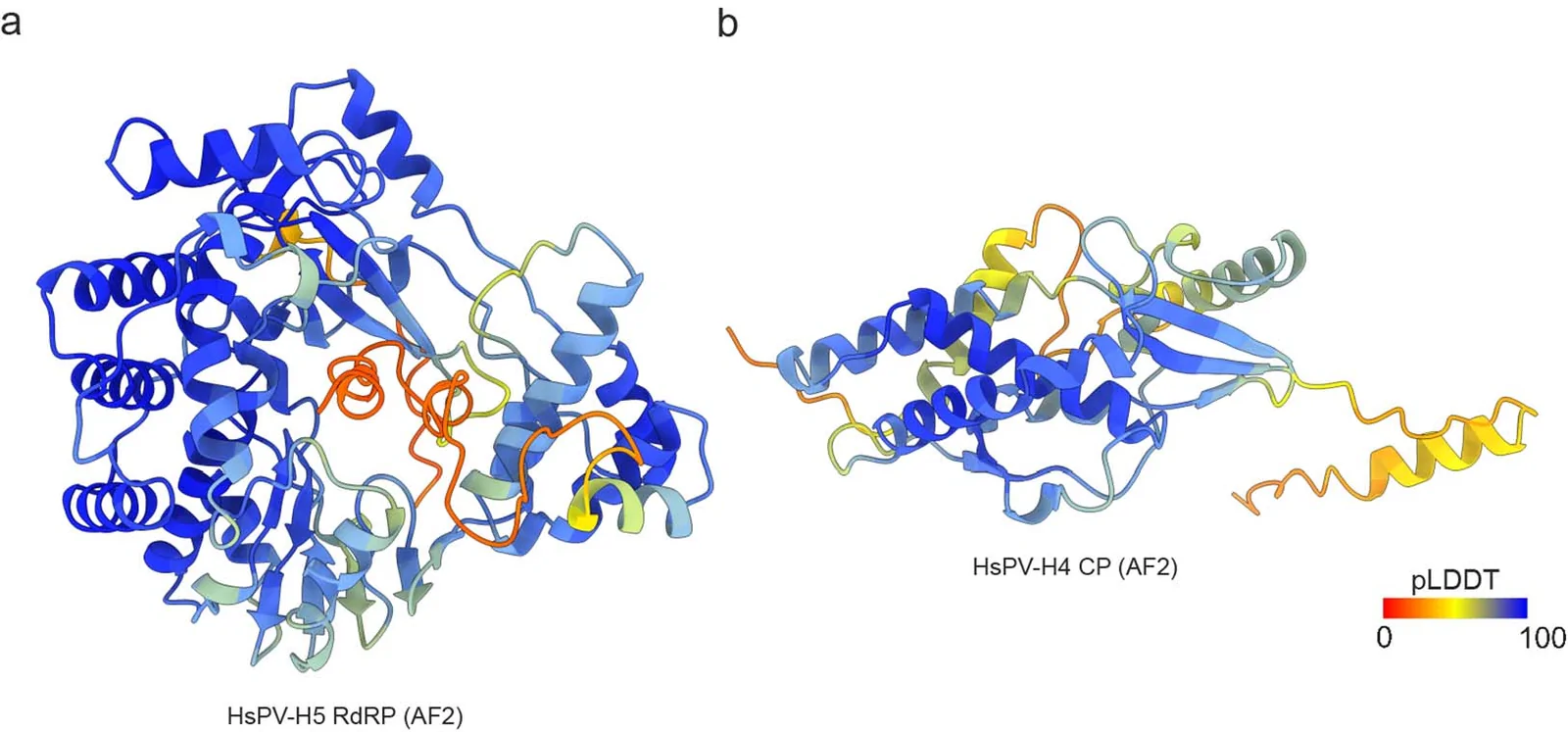
pLDDT scores of HsPV RdRP and CP. Quality assessment of the AF2 model of the HsPV a, RdRP and b, CP. The structural model is colored based on the pLDDT scores, with the color key shown at the bottom right corner.

Analysis of the FLDS RNA sequencing data from the stations H4 (68.8 °C, pH 3.2), H5 (69.7 °C, pH 3.1) and Y66 (68.7 °C, pH 2.7) revealed a bipartite virus genome unrelated to HsRV (Fig. 5a, Extended Data Table 2 and Fig. 2b). The genomic segments, RNA1 and RNA2, shared conserved 5′ terminal sequences and encoded one and two proteins, respectively (Fig. 5b). ORF1 of RNA1 was unambiguously identified as an RdRP, yielding significant BLASTP hits to RdRPs of members of the *Partitiviridae* family, with the best hit being to the unclassified Driatsky virus (QIS87951; *E*-value = 1 × 10^−95^). We denoted this virus as hot spring partiti-like virus (HsPV). The similarity between the termini of the segments precluded assignment of all sets of segments to particular virus strains. However, on the basis of co-occurrence in the same sample and similar abundances, segment pairs RNA1_a and RNA2_b from sample H5 could be assigned to the same virus strain, HsPV1. Phylogenetic analysis of the RdRP sequence from diverse classified and unclassified partiti-like viruses showed that HsPVs and Driatsky virus (see below) were nested within genPartiti.0029 (Fig. 5c), a highly diverse, unclassified group defined in a recent metatranscriptome study^8^. The genPartiti.0029, including HsPV and Driatsky virus and several other subclades, formed a deep clade separate from all other partitiviruses. Thus, genPartiti.0029 can be considered a separate sister family to the bona fide *Partitiviridae*. AF2 modelling yielded an HsPV RdRP model closely similar to that of the RdRP of the deltapartitivirus pepper cryptic virus 1 (PCV1; Fig. 5d and Extended Data Fig. 3a), which was confirmed by DALI *Z*-score-based clustering (Fig. 4a), where the two viruses formed a clade next to picobirnaviruses.

**Extended Data Fig. 4:**
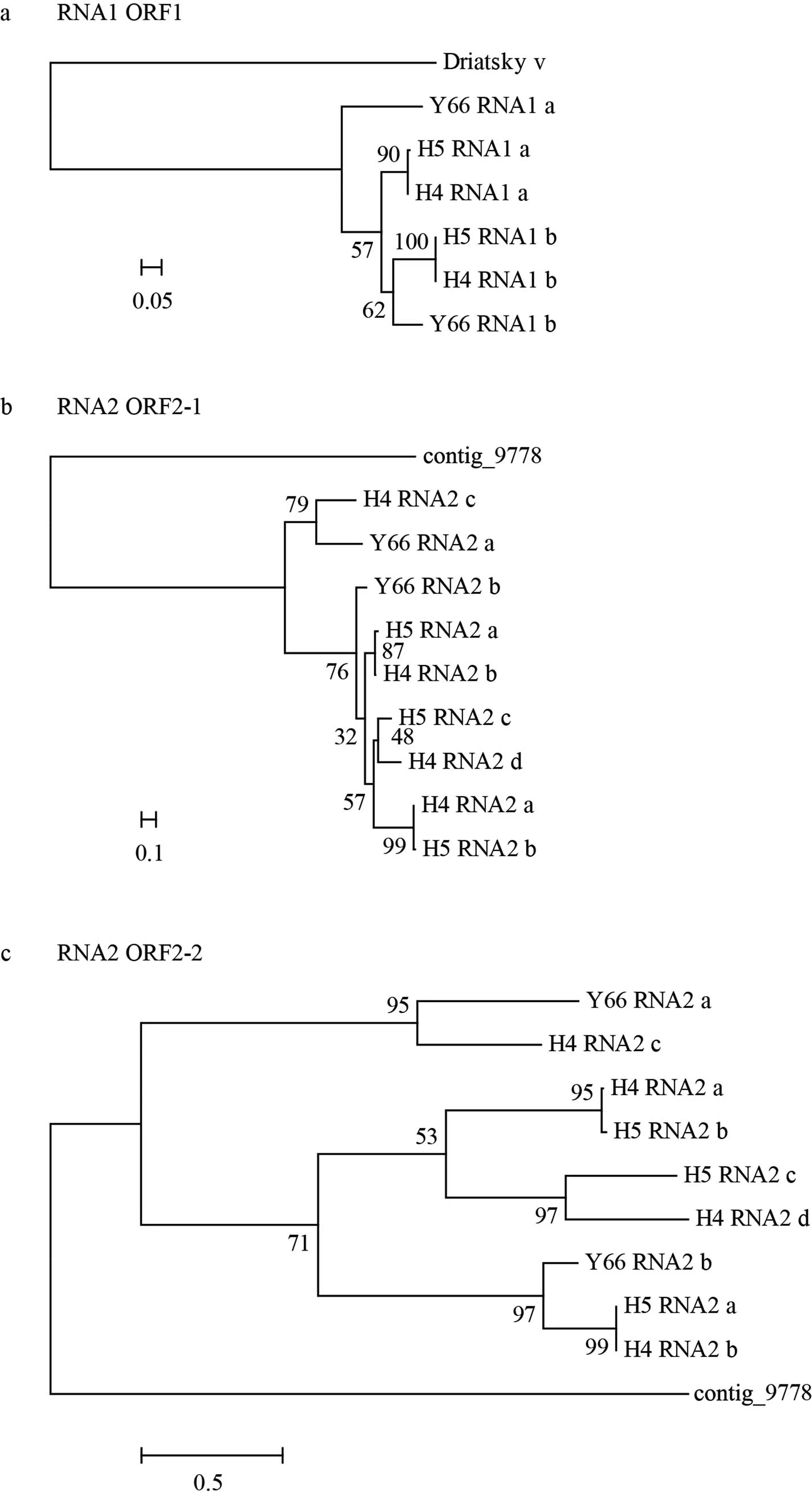
HsPV phylogeny. Maximum-likelihood trees of each ORF encoded by HsPVs and related sequences. Sequences were aligned using MEGA6. The ambiguous positions in the alignment were removed using TrimAl. The maximum likelihood tree was constructed using RAxML. The best-fitting substitution model was selected by ProtTest. Numbers indicate the percentage bootstrap support from 1,000 RAxML bootstrap replicates. We used RAxML with the **a**, LG+G+I+F model for ORF1, **b**, LG+G model for ORF2-1 and **c**, LG+G+I+F model for ORF2-2.

Structural modelling of RNA2 ORF1 of different HsPV strains and Driatsky virus yielded a high-quality model (pLDDT = 78.8), with only the terminal regions being of lower quality (Extended Data Fig. 3b and Supplementary Text). Structure similarity searches against the PDB database using DALI produced significant hits to capsid proteins (CPs) of partitiviruses and picobirnaviruses^26–28^, with the best match (*Z*-score = 8.2) to the CP of PCV1 (Fig. 5e; PDB ID: 7ncr; *Deltapartitivirus*). Thus, the RdRP phylogeny and structural similarity of the CPs indicate that HsPV is related to members of the family *Partitiviridae*. The phylogenetic relationship between amino acid sequences of HsPVs is shown in Extended Data Fig. 4.

### HsRV and HsPV probably infect prokaryotic hosts

All samples in which HsRV and HsPV were detected nearly exclusively contained rRNA sequences from prokaryotes, with eukaryotic presence being below 1%. This is consistent with eukaryotes being unable to thrive in polyextremophilic conditions combining high temperatures and acidic pH. The microbial communities in all 4 samples (H4, H5, Y66 and Oi) were dominated by bacteria (Supplementary Text). Thus, HsRV and HsPV most probably infect bacteria. To test this inference, we predicted ribosome-binding SD motifs in all HsRV and HsPV strains. SD motifs are essential for translation initiation in many prokaryotes, and their conservation is a diagnostic feature of prokaryotic genes that has been used to assign bacterial hosts to several groups of RNA viruses, namely, picobirnaviruses and partitiviruses, including genPartiti.0019 and genPartiti.0029 (refs. ^8,29^). Analysis of the HsRV and HsPV genomes showed that nearly every gene in these viruses is preceded by an SD motif (Figs. 2a, 3a and 5a and Extended Data Table 6), further suggesting that both HsRV and HsPV infect prokaryotic hosts. Bacteria of the genus *Hydrogenobaculum* (family *Aquificaceae*) were predominant (>95%) in samples H4 and H5 and highly abundant in Y66 (>85%), suggesting that HsPV detected in all three samples infects *Hydrogenobaculum* sp.

No CRISPR spacers matching the HsRV and HsPV genomes were identified in the public databases or the 919 CRISPR spacer sequences obtained by metagenomic DNA sequencing of the hot spring samples (Supplementary Text). Nevertheless, the lack of eukaryotes in the hot spring samples, contrasted by the dominance of bacteria, together with the presence of typical prokaryotic SD motifs upstream of the predicted virus genes and the polycistronic organization of the viral genomes, strongly suggest that HsRV and HsPV are viruses of thermophilic bacteria.

## Discussion

The discovery of the HsRV-like group of riboviruses recapitulates previous findings of several small groups of riboviruses that are predicted to infect bacteria and might become distinct phyla^7,8^. However, the RdRPs of HsRV and its relatives seem to deviate from the RdRP consensus farther than any of the other recently discovered putative phyla, with none of which they appear to be affiliated, and possess unusual (predicted) structural features that appear to link them to viral RTs. Whether this connection reflects an intermediate position of the HsRV-like viruses between the kingdoms *Orthornavirae* and *Pararnavirae*, or results from convergent evolution, remains uncertain and should be clarified by sequencing and structural analysis of additional members of this group, or possibly, other groups of riboviruses with similar features. Furthermore, although we did not detect any evidence of the formation of DNA copies of the genomes of HsRV-like viruses, it will be of interest to determine whether their RdRPs possess RT activity, as shown for some viral RdRPs^30^. Regardless, HsRV-like viruses are strong candidates for a separate phylum in the kingdom *Orthornavirae*, which we propose to name ‘*Artimaviricota*’ after the potential link to viral RTs (arti) and ‘artima’ which means ‘close’ in Lithuanian, or even a third kingdom within the realm *Riboviria*.

This report is a proof of concept for the discovery of multiple, perhaps many groups of riboviruses with unexpected properties by obtaining complete genomes of segmented riboviruses from meta-dsRNA-seq data and mining metatranscriptomes from habitats with distinct conditions. Information on non-RdRP segments is unavailable for most of the RNA virus lineages identified only from metatranscriptomes, whereas riboviruses that are distantly related to known RNA viruses can be missed altogether. Our approach helps to overcome these limitations and contributes to a more complete characterization of RNA viromes.

## Methods

### Sample collection

A total of 11 samples were collected from five hot springs regions in southern Japan, in proximity to active volcanoes (Table 1 and Supplementary Text), according to the instructions of Unzen City, Unzen Nature Conservation Bureau and private companies that maintain each hot spring region. Temperature, pH and dissolved oxygen (DO) were measured in situ by using a multiple electrode sensor (D-55, Horiba). H_2_S concentration was calculated from the spectrophotometric absorbance at 680 nm of methylene blue formed from a reaction with *N*,*N*-dimethyl-*p*-phenylenediamine in FeCl_2_-HCl solution. Typical measurement errors are 0.1 for pH, 0.1 mg l^−1^ for DO and 5% for H_2_S. Dissolved chemicals and water isotope ratios of the geothermal waters were also measured and are summarized in Supplementary Text.

**Table 1:** Characteristics of hot spring water samples

Code	Geographical coordinates	Area	Temp (°C)	pH	DO (mg l^−1^)	H_2_S (mM)	Sampling date	Site characteristics
H4	31° 54′ 07.5″ N, 130° 50′ 06.2″ E	Hayashida	68.8	3.2	2.1	1.3	10 Mar 2017	Transparent water pool with sulfur precipitates
H5	31° 54′ 07.5″ N, 130° 50′ 06.2″ E	Hayashida	69.7	3.1	2.0	1.8	10 Mar 2017	Transparent water pool with sulfur precipitates
T1	31° 54′ 37.7″ N, 130° 49′ 00.6″ E	Tearai	92.1	2.9	−	0.0	09 Mar 2017	Yellowish grey water pool with active venting
T2		Tearai	95.9	2.1	−	0.0	09 Mar 2017	Yellowish grey vent pool
T3		Tearai	94.4	2.4	0.0	0.0	09 Mar 2017	Slightly grey water vent pool
T4		Tearai	92.8	2.7	0.0	0.0	09 Mar 2017	Yellowish grey water vent pool
Y66	31° 55′ 03.8″ N, 130° 48′ 40.4″ E	Yunoike	68.7	2.7	2.1	0.0	10 Mar 2017	Yellowish grey vent pool
Y80		Yunoike	75–86^a^	2.5	1.5	0.0	10 Mar 2017	Muddy small vent pool
Y86		Yunoike	86.5	2.5	0.0	0.0	10 Mar 2017	Muddy boiling vent pool
Oi	32° 44′ 25.3″ N, 130° 15′ 48.4″ E	Unzen	79.3	2.2	0.0	0.4	18 Nov 2015	Yellowish grey vent pool
Ob	32° 43′ 33.0″ N, 130° 12′ 24.7″ E	Obama	72.8	7.9	0.0	0.0	17 Nov 2015	Transparent water pool under hot spring water tank

^a^There were temperature gradients in the pool site: surface layer 75.0 °C; bottom layer 81.6 °C, 80.3 °C, 85.9 °C; middle layer 81.0 °C.

Most of the sampling sites were characterized by high temperatures above 65 °C, acidic pH (2–3, except for Site Ob with a slightly alkaline pH of 7.9) and lower level of DO with accompanying grey mud or light-yellow sulfur deposits. At each sampling station, ∼10 l of hot spring water was collected in a sterilized plastic bag and then filtered with 0.2‐μm‐pore‐size cellulose acetate membrane filters in 47 mm diameter (Advantecn) within 0.5–3 h after sampling. The filters were stored at −80 °C until nucleic acid extraction.

### RNA extraction

Cells collected on a portion of the 0.2‐μm‐pore‐size filters corresponding to ∼2 l of hot spring water were pulverized in a mortar in liquid nitrogen and suspended in dsRNA extraction buffer (20 mM Tris–HCl, pH 6.8, 200 mM NaCl, 2 mM EDTA, 1% SDS and 0.1% (v/v) β‐mercaptoethanol) or TRIzol buffer for ds- and ssRNA purification, respectively. For dsRNA purification, total nucleic acids were manually extracted with SDS-phenol. dsRNA was purified using the cellulose resin chromatography method^16,31^. The remaining DNA and ssRNA were removed by DNase I (Invitrogen) and S1 nuclease (Invitrogen) treatment^19^. For ssRNA purification, the ssRNA fraction was collected using the TRIzol Plus RNA purification kit (Invitrogen) according to manufacturer protocol. The ssRNA fraction was treated with DNase I (Invitrogen) and concentrated using the RNA Clean and Concentrator-5 kit (Zymoresearch).

### Complementary DNA synthesis

Complementary DNA (cDNA) was synthesized from purified dsRNA and ssRNA as described previously^19^. In brief, purified dsRNA was physically fragmented into ∼1.5 kbp and adapter oligonucleotide (U2: 5′-GAC GTA AGA ACG TCG CAC CA-3′) was ligated to the 3′-end of fragmented dsRNAs. After heat denaturation with an oligonucleotide primer (U2-comp: 5′-TGG TGC GAC GTT CTT ACG TC-3′), that has complementary sequence to the adapter oligonucleotide, cDNA was synthesized using SMARTer RACE 5′/3′ kit (Takara Bio). ssRNA was converted into cDNA using SMARTer Universal Low Input RNA kit according to manufacturer protocol (Takara Bio). After PCR amplification, cDNA was fragmented by a Covaris S220 ultrasonicator.

### Illumina sequencing library construction and sequencing

Illumina sequencing libraries were then constructed using KAPA Hyper Prep Kit Illumina platforms (Kapa Biosystems) from the physically shared environmental cDNAs. The libraries were sequenced using the Illumina MiSeq v3 Reagent kit (600 cycles) with 300-bp paired-end reads on the Illumina MiSeq platform.

### Data processing

Trimmed reads were obtained using a custom Perl pipeline script (https://github.com/takakiy/FLDS) from dsRNA raw sequence reads^17^. The clean reads were subjected to de novo assembly using CLC GENOMICS WORKBENCH v.11.0 (Qiagen) with the following parameters: a minimum contig length of 500, word value set to auto and bubble size set to auto. The full-length sequences were manually extracted using CLC GENOMICS WORKBENCH v.11.0 (Qiagen), Genetyx v.14 (Genetyx) and Tablet viewer v.1.19.09.03 (ref. ^32^) as described previously^33^. In brief, contigs for which both termini were determined to be the ends were identified as full‐length sequences. In cases of dominant reads (more than 10 reads) that stopped in the same position around the ends of contigs in the mapping analysis, that position was recognized as the segment (genome) end. In this study, major full-length sequences with >1,000 average coverage were analysed, except for the Oi sample where all full-length sequences were recovered. From ssRNA raw sequence reads, trimmed reads were also obtained using a custom Perl pipeline script (https://github.com/takakiy/FLDS). The resultant clean reads were applied to phyloFlash^20^ to identify active microbes in our samples.

### Sequence analyses

RNA viral genes were identified using the BLASTX programme against the NCBI non-redundant (nr) database with an *E*-value ≤ 1 × 10^−05^. The ribosome-binding SD motifs were identified using Prodigal^34^. Remote homology searches were performed using HHpred against the PDB70, Pfam, UniProt-SwissProt-viral70 and NCBI-CD (conserved domains) databases^35^. MSA of HsRV RNA1_ORF4s was built using MEGA6 (ref. ^36^). The alignment was then used as input in HHblits 3.3.0, which compared the alignments to the PDB70 (pdb70_from_mmcif_220313) database. Transmembrane domains were predicted using TMHMM^37^.

### Search for HsRV homologues in public databases

To identify viruses related to HsRV in the IMG/VR database^38^, BLASTP searches (*E*-value ≤ 1 × 10^−05^) queried with the RdRP sequences encoded by HsRV-like contigs previously deposited to GenBank (accessions: BDQA01000957 and BDQA01004869) were performed on the IMG/VR website (https://img.jgi.doe.gov/cgi-bin/vr/main.cgi?section=Viral&page=findViralGenesBlast). The nucleotide sequences of the contigs encoding the related RdRPs were downloaded and annotated as described above for the HsRVs.

### Modelling protein structures with AlphaFold2 and structural comparisons

Structural predictions for HsRV and HsRV-like RdRP amino acid sequences were performed using ColabFold 1.5.1 installed locally through LocalColabFold (https://github.com/YoshitakaMo/localcolabfold). A custom MSA with ten HsRV (HsRV_La∼d, H5_contig_1, Oi_contig_1, Oi_contig_3, Oi_contig_5, Oi_contig_8, Oi_contig_9) and five HsRV-like (BDQA01000957, BDQA01004869, Ga0456180, Ga0393213, Ga0169446) RdRP amino acid sequences was used as input. The number of recycles used for HsRV_La ORF4 and HsRV-like RdRP predictions were 6 and 10, respectively. For the core (motifs A–C) region of marine HsRV-like RdRP BDQA01004869 (Fig. 3d), 20 recycles were used. For Fig. 4a, Ambiviricota RdRP model (pLDDT 95, predicted template modeling (pTM) score 0.938) was generated with 3 recycles using a custom MSA of 422 Ambivirus RdRP sequences available at https://github.com/ababaian/serratus/wiki/ambivirus_extended_data (ref. ^24^). Paraxenoviricota (TARA_132_DCM_0.22-3_k119_33585_1_799) RdRP model (pLDDT 88.6, pTM 0.882) was generated with 20 recycles using a custom MSA of 12 amino acid sequences obtained by running BLASTP against ORFs from 44779_RdRP_contigs available at https://datacommons.cyverse.org/browse/iplant/home/shared/iVirus/ZayedWainainaDominguez-Huerta_RNAevolution_Dec2021/Contigs (ref. ^7^). Similarly, Wamoviricota (84SUR2MMQQ14_2_ERR1712161_contig_61452_3_468) RdRP model (pLDDT 84.5, pTM 0.822) was modelled with 20 recycles using a custom MSA of 6 sequences from the 44779_RdRP_contigs^7^ and 56 additional sequences obtained from a BLASTP search against the IMG/VR database. p.0002 (ND_055403_2847-982) RdRP model (pLDDT 84.3, pTM 0.864) was generated with 12 recycles using a custom MSA with 107 p.0002 RdRP sequences kindly provided by Dr Yuri I. Wolf^8^. The RdRPs of HsPV-H5 and PCV1 (GenBank ID: YP_009466859) were modelled using AlphaFold 2 through ColbFold (v.1.5.2)^39,40^ with 6 recycles each. For the HsPV-H4 CP modelling, an alignment of RNA2 ORF1 homologues from HsPV-like viruses and Driatsky virus was used as a template with 12 recycles. The obtained model had a medium quality (average pLDDT = 57.3), although the central region was modelled with higher quality (average pLDDT > 70). This model was used as a query in DALI search, which identified the CP of PCV1 (PDB ID: 7ncr) as the best hit with a *Z*-score of 6.5. Thus, to improve the quality of the HsPV-H4 CP model, we repeated the modelling using the same sequence alignment and providing the PDB structure of the PCV1 CP as a template, with 24 recycles. The obtained model had an average pLDDT score of 78.1. Model display, structural alignment, colouring and figure preparation were performed using UCSF ChimeraX software^41^.

### Phylogenetic analysis

Amino acid sequences of RdRP encoded by identified viruses and viruses related to the family *Partitiviridae* were aligned using MAFFT (G-INS-1)^42^. The ambiguous positions in the alignment were removed using TrimAl (gap threshold 0.2)^43^. The maximum-likelihood tree was constructed using IQ-TREE (v.2.0.6)^44^. The best-fitting substitution model was selected using ModelFinder^45^ and was LG + F + R8. Node supports were estimated using the SH-like approximate likelihood-ratio test (SH-aLRT) with 1,000 replicates. For phylogenetic analysis of the HsRV-like RdRPs, the proteins were aligned using PROMALS3D^46^ and uninformative positions we removed using TrimAl with the gappyout functions^43^. The final alignment contained 520 positions. The maximum-likelihood tree was constructed using IQ-TREE (v.2.0.6)^44^. The best-fitting substitution model was selected using ModelFinder^45^ and was LG + I + G4. Node supports were estimated using SH-aLRT (1,000 replicates).

### Reporting summary

Further information on research design is available in the Nature Portfolio Reporting Summary linked to this article.

## Supplementary information

## Figures and Tables

**Extended Data Table 1:**
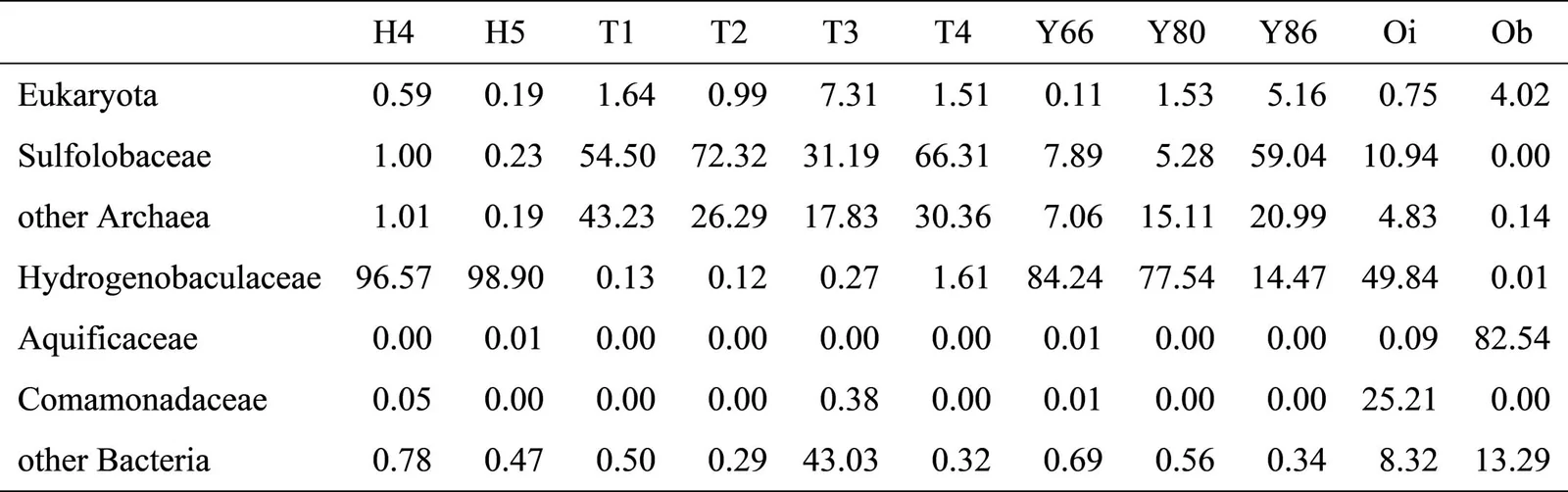
Relative abundances of rRNA reads in ssRNA seq of representative microbial lineages

**Extended Data Table 2:**
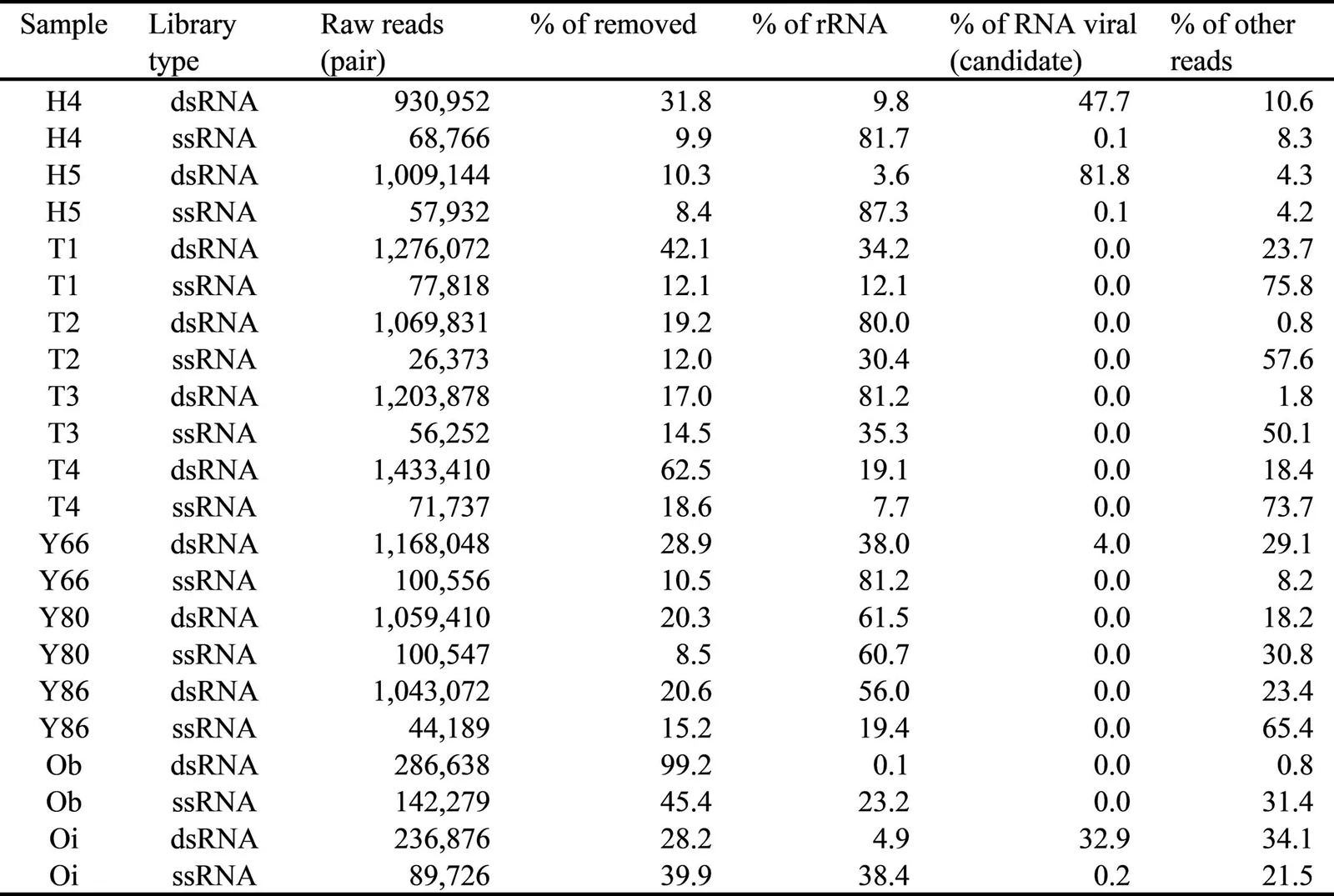
Classification of NGS reads

**Extended Data Table 3:**
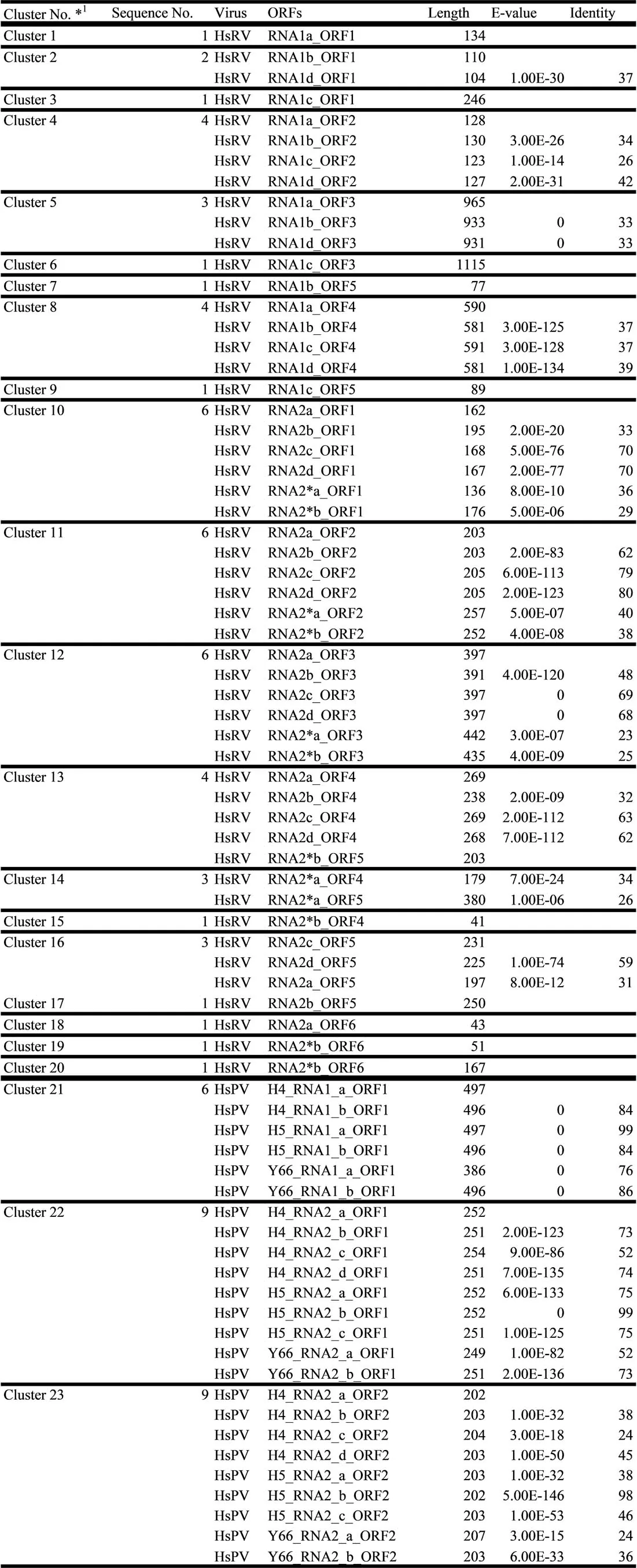
Result of CDS clustering using a standard BLAST-mcl pipeline

^*1^The CDSs were clustered using a standard BLAST-mcl pipeline [BLASTP (v2.9.0) with default options, hits selected based on E-value < 1e-10, MCL clustering (v.14-137) with an inflation value of 2.8].

**Extended Data Table 4:**
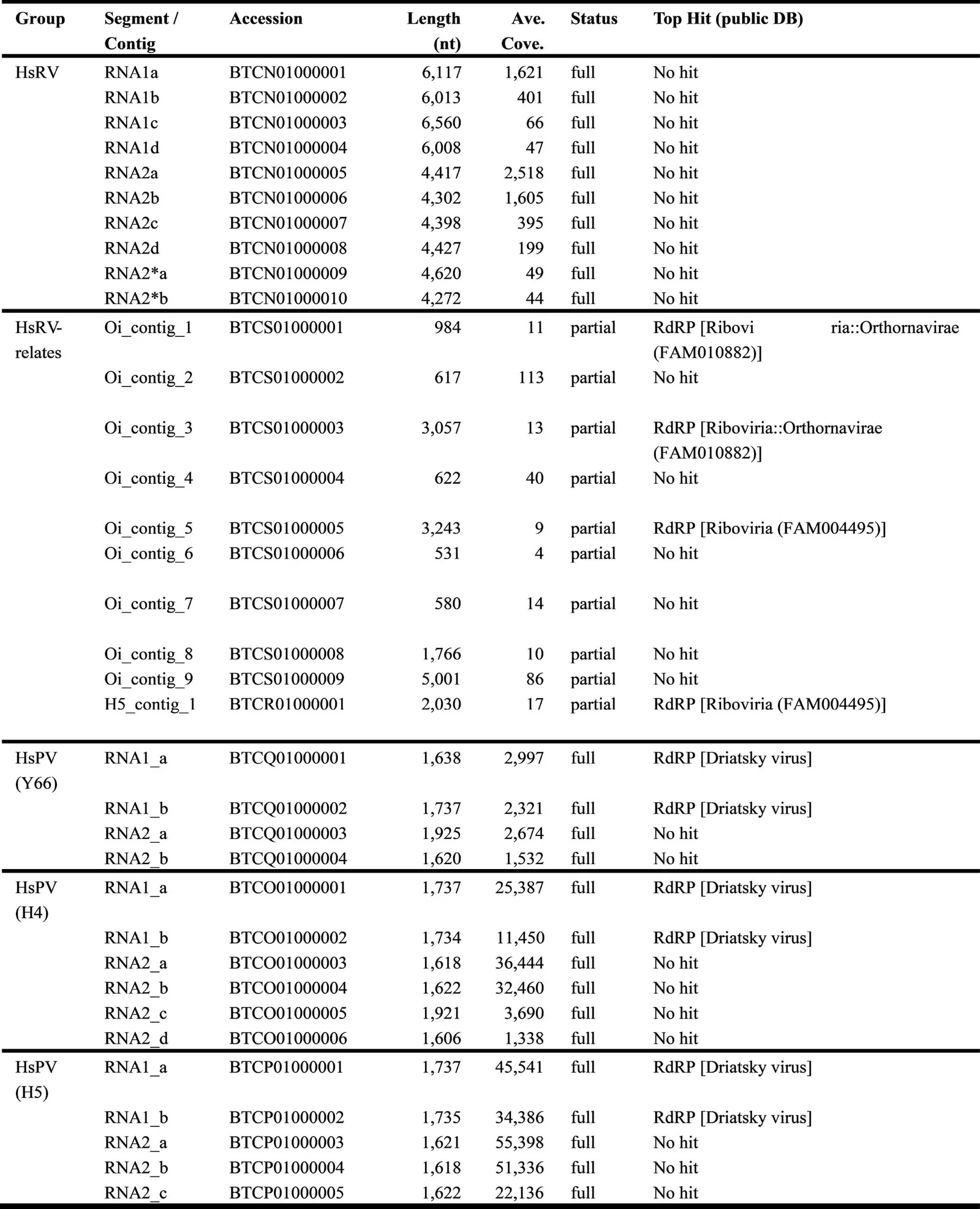
RNA virus and virus-like genomes identified in this study

**Extended Data Table 5:**
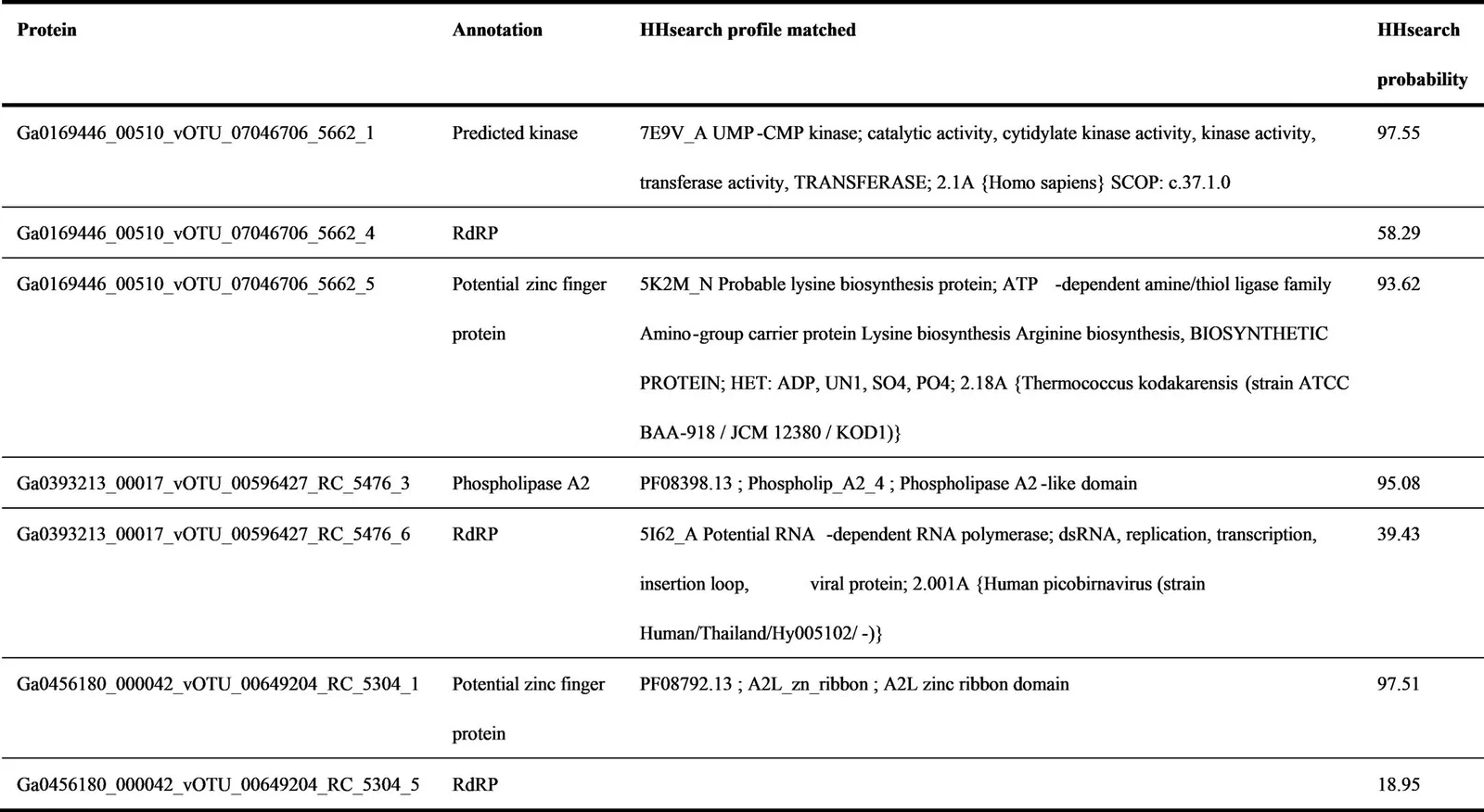
HHsearch hits for the IMG/VR virus proteins

**Extended Data Table 6:**
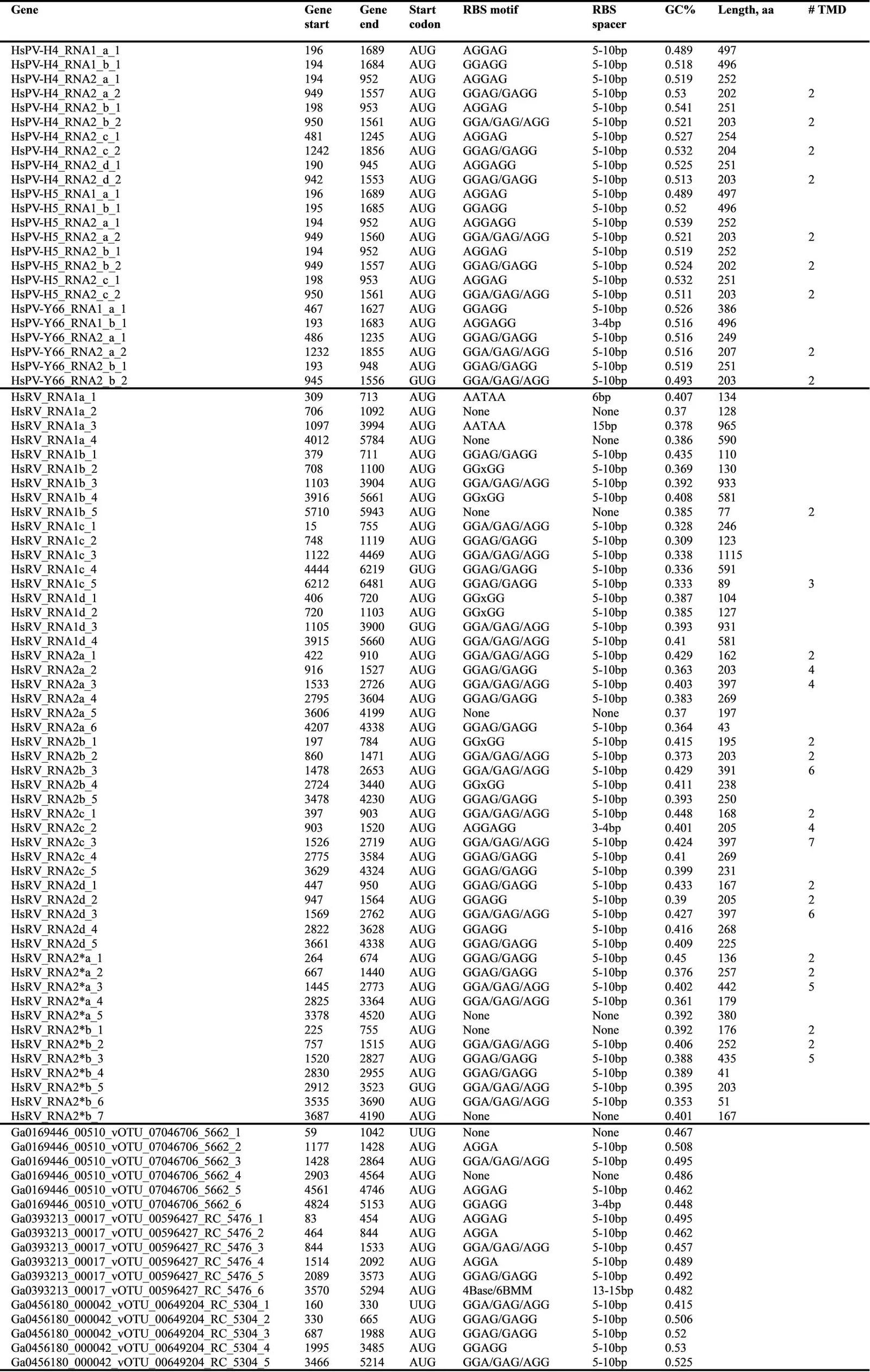
Detected RBS motif

## Data Availability

Datasets obtained in this study have been made available in the GenBank database repository (accession nos. HsRV: BTCN01000001–BTCN01000010; HsPV-H4: BTCO01000001–BTCO01000006; HsPV-H5: BTCP01000001–BTCP01000005; HsPV-Y66: BTCQ01000001–BTCQ01000004; H5_contig_1: BTCR01000001; Oi_contig_1-9: BTCS01000001–BTCS01000009) and Short Read Archive database (accession no. DRA016131). Datasets (PDB70 mmcif_2023-10-24, Pfam v.35, UniProt-SwissProt-viral70_Nov_2021 and NCBI-CD v.3.19) are available at http://ftp.tuebingen.mpg.de/pub/protevo/toolkit/databases/hhsuite_dbs/. Searches using the IMG/VR dataset were available only at https://img.jgi.doe.gov/cgi-bin/vr/main.cgi?section=WorkspaceBlast&page=viralform. Datasets (SILVA SSU v.138, Neo-HMM v.1.1 and RVDB-HMM v.23.0) are publicly available.

## Code availability

A custom code used in this study has been made available in a git repository publicly available on GitHub at https://github.com/takakiy/FLDS (Cleanup_FLDS.pl).

## References

[1] Simmonds, P. et al. Consensus statement: virus taxonomy in the age of metagenomics. *Nat. Rev. Microbiol.* **15**, 161–168 (2017). 28134265 10.1038/nrmicro.2016.177

[2] Shi, M. et al. Redefining the invertebrate RNA virosphere. *Nature* **540**, 539–543 (2016). 27880757 10.1038/nature20167

[3] Wolf, Y. I. et al. Origins and evolution of the global RNA virome. *mBio* **9**, e02329-18 (2018). 30482837 10.1128/mBio.02329-18 PMC6282212

[4] Wolf, Y. I. et al. Doubling of the known set of RNA viruses by metagenomic analysis of an aquatic virome. *Nat. Microbiol.* **5**, 1262–1270 (2020). 32690954 10.1038/s41564-020-0755-4 PMC7508674

[5] Shi, M., Zhang, Y. Z. & Holmes, E. C. Meta-transcriptomics and the evolutionary biology of RNA viruses. *Virus Res.* **243**, 83–90 (2018). 29111455 10.1016/j.virusres.2017.10.016 PMC7127328

[6] Shi, M. et al. The evolutionary history of vertebrate RNA viruses. *Nature* **556**, 197–202 (2018). 29618816 10.1038/s41586-018-0012-7

[7] Zayed, A. A. et al. Cryptic and abundant marine viruses at the evolutionary origins of Earth’s RNA virome. *Science* **376**, 156–162 (2022). 35389782 10.1126/science.abm5847 PMC10990476

[8] Neri, U. et al. Expansion of the global RNA virome reveals diverse clades of bacteriophages. *Cell* **185**, 4023–4037 (2022). 36174579 10.1016/j.cell.2022.08.023

[9] Edgar, R. C. et al. Petabase-scale sequence alignment catalyses viral discovery. *Nature* **602**, 142–147 (2022). 35082445 10.1038/s41586-021-04332-2

[10] Koonin, E. V. et al. Global organization and proposed megataxonomy of the virus world. *Microbiol. Mol. Biol. Rev.* **84**, e00061-19 (2020). 32132243 10.1128/MMBR.00061-19 PMC7062200

[11] Koonin, E. V., Dolja, V. V. & Krupovic, M. Origins and evolution of viruses of eukaryotes: the ultimate modularity. *Virology* **479-480**, 2–25 (2015). 25771806 10.1016/j.virol.2015.02.039 PMC5898234

[12] Nasir, A., Forterre, P., Kim, K. M. & Caetano-Anolles, G. The distribution and impact of viral lineages in domains of life. *Front. Microbiol.* **5**, 194 (2014). 24817866 10.3389/fmicb.2014.00194 PMC4012193

[13] Callanan, J. et al. Leviviricetes: expanding and restructuring the taxonomy of bacteria-infecting single-stranded RNA viruses. *Microb. Genomics* **7**, 000686 (2021). 10.1099/mgen.0.000686 PMC8743537 34747690

[14] Callanan, J. et al. Expansion of known ssRNA phage genomes: from tens to over a thousand. *Sci. Adv.* **6**, eaay5981 (2020). 32083183 10.1126/sciadv.aay5981 PMC7007245

[15] Krishnamurthy, S. R., Janowski, A. B., Zhao, G., Barouch, D. & Wang, D. Hyperexpansion of RNA bacteriophage diversity. *PLoS Biol.* **14**, e1002409 (2016). 27010970 10.1371/journal.pbio.1002409 PMC4807089

[16] Morris, T. J. & Dodds, J. A. Isolation and analysis of double-stranded-RNA from virus-infected plant and fungal tissue. *Phytopathology* **69**, 854–858 (1979). 10.1094/Phyto-69-854 40934407

[17] Hirai, M. et al. RNA viral metagenome analysis of subnanogram dsRNA using fragmented and primer ligated dsRNA sequencing (FLDS). *Microbes Environ.* **36**, ME20152 (2021). 33952860 10.1264/jsme2.ME20152 PMC8209451

[18] Urayama, S., Takaki, Y. & Nunoura, T. FLDS: a comprehensive dsRNA sequencing method for intracellular RNA virus surveillance. *Microbes Environ.* **31**, 33–40 (2016). 26877136 10.1264/jsme2.ME15171 PMC4791113

[19] Urayama, S. et al. Unveiling the RNA virosphere associated with marine microorganisms. *Mol. Ecol. Resour.* **18**, 1444–1455 (2018). 30256532 10.1111/1755-0998.12936

[20] Gruber-Vodicka, H. R., Seah, B. K. & Pruesse, E. phyloFlash: rapid small-subunit rRNA profiling and targeted assembly from metagenomes. *mSystems* **5**, e00920 (2020). 33109753 10.1128/mSystems.00920-20 PMC7593591

[21] Yang, Y. et al. Characterization of the first double-stranded RNA bacteriophage infecting *Pseudomonas aeruginosa*. *Sci. Rep.* **6**, 38795 (2016). 27934909 10.1038/srep38795 PMC5146939

[22] Venkataraman, S., Prasad, B. & Selvarajan, R. RNA dependent RNA polymerases: insights from structure, function and evolution. *Viruses* **10**, 76 (2018). 29439438 10.3390/v10020076 PMC5850383

[23] Te Velthuis, A. J. Common and unique features of viral RNA-dependent polymerases. *Cell. Mol. Life Sci.* **71**, 4403–4420 (2014). 25080879 10.1007/s00018-014-1695-z PMC4207942

[24] Forgia, M. et al. Hybrids of RNA viruses and viroid-like elements replicate in fungi. *Nat. Commun.* **14**, 2591 (2023). 37147358 10.1038/s41467-023-38301-2 PMC10162972

[25] Krupovic, M. et al. Ortervirales: new virus order unifying five families of reverse-transcribing viruses. *J. Virol.* **92**, e00515–18 (2018). 29618642 10.1128/JVI.00515-18 PMC5974489

[26] Luque, D., Mata, C. P., Suzuki, N., Ghabrial, S. A. & Castón, J. R. Capsid structure of dsRNA fungal viruses. *Viruses* **10**, 481 (2018). 30205532 10.3390/v10090481 PMC6164181

[27] Byrne, M., Kashyap, A., Esquirol, L., Ranson, N. & Sainsbury, F. The structure of a plant-specific partitivirus capsid reveals a unique coat protein domain architecture with an intrinsically disordered protrusion. *Commun. Biol.* **4**, 1155 (2021). 34615994 10.1038/s42003-021-02687-w PMC8494798

[28] Duquerroy, S. et al. The picobirnavirus crystal structure provides functional insights into virion assembly and cell entry. *EMBO J.* **28**, 1655–1665 (2009). 19407816 10.1038/emboj.2009.109 PMC2693148

[29] Krishnamurthy, S. R. & Wang, D. Extensive conservation of prokaryotic ribosomal binding sites in known and novel picobirnaviruses. *Virology* **516**, 108–114 (2018). 29346073 10.1016/j.virol.2018.01.006

[30] Peyambari, M., Guan, S. & Roossinck, M. J. RdRp or RT, that is the question. *Mol. Biol. Evol.* **38**, 5082–5091 (2021). 34352104 10.1093/molbev/msab235 PMC8557412

[31] Okada, R., Kiyota, E., Moriyama, H., Fukuhara, T. & Natsuaki, T. A simple and rapid method to purify viral dsRNA from plant and fungal tissue. *J. Gen. Plant Pathol.* **81**, 103–107 (2015).

[32] Milne, I. et al. Tablet—next generation sequence assembly visualization. *Bioinformatics* **26**, 401–402 (2010). 19965881 10.1093/bioinformatics/btp666 PMC2815658

[33] Urayama, S., Takaki, Y., Hagiwara, D. & Nunoura, T. dsRNA-seq reveals novel RNA virus and virus-like putative complete genome sequences from *Hymeniacidon* sp. sponge. *Microbes Environ.* **35**, ME19132 (2020). 32115438 10.1264/jsme2.ME19132 PMC7308569

[34] Hyatt, D. et al. Prodigal: prokaryotic gene recognition and translation initiation site identification. *BMC Bioinformatics* **11**, 119 (2010). 20211023 10.1186/1471-2105-11-119 PMC2848648

[35] Gabler, F. et al. Protein sequence analysis using the MPI bioinformatics toolkit. *Curr. Protoc. Bioinformatics* **72**, e108 (2020). 33315308 10.1002/cpbi.108

[36] Tamura, K., Stecher, G., Peterson, D., Filipski, A. & Kumar, S. MEGA6: molecular evolutionary genetics analysis version 6.0. *Mol. Biol. Evol.* **30**, 2725–2729 (2013). 24132122 10.1093/molbev/mst197 PMC3840312

[37] Krogh, A., Larsson, B., von Heijne, G. & Sonnhammer, E. L. Predicting transmembrane protein topology with a hidden Markov model: application to complete genomes. *J. Mol. Biol.* **305**, 567–580 (2001). 11152613 10.1006/jmbi.2000.4315

[38] Camargo, A. P. et al. IMG/VR v4: an expanded database of uncultivated virus genomes within a framework of extensive functional, taxonomic, and ecological metadata. *Nucleic Acids Res.* **51**, D733–D743 (2023). 36399502 10.1093/nar/gkac1037 PMC9825611

[39] Mirdita, M. et al. ColabFold: making protein folding accessible to all. *Nat. Methods* **19**, 679–682 (2022). 35637307 10.1038/s41592-022-01488-1 PMC9184281

[40] Jumper, J. et al. Highly accurate protein structure prediction with AlphaFold. *Nature* **596**, 583–589 (2021). 34265844 10.1038/s41586-021-03819-2 PMC8371605

[41] Pettersen, E. F. et al. UCSF ChimeraX: structure visualization for researchers, educators, and developers. *Protein Sci.* **30**, 70–82 (2021). 32881101 10.1002/pro.3943 PMC7737788

[42] Katoh, K. & Standley, D. M. MAFFT multiple sequence alignment software version 7: improvements in performance and usability. *Mol. Biol. Evol.* **30**, 772–780 (2013). 23329690 10.1093/molbev/mst010 PMC3603318

[43] Capella-Gutiérrez, S., Silla-Martínez, J. M. & Gabaldón, T. trimAl: a tool for automated alignment trimming in large-scale phylogenetic analyses. *Bioinformatics* **25**, 1972–1973 (2009). 19505945 10.1093/bioinformatics/btp348 PMC2712344

[44] Minh, B. Q. et al. IQ-TREE 2: new models and efficient methods for phylogenetic inference in the genomic era. *Mol. Biol. Evol.* **37**, 1530–1534 (2020). 32011700 10.1093/molbev/msaa015 PMC7182206

[45] Kalyaanamoorthy, S., Minh, B. Q., Wong, T. K., von Haeseler, A. & Jermiin, L. S. ModelFinder: fast model selection for accurate phylogenetic estimates. *Nat. Methods* **14**, 587–589 (2017). 28481363 10.1038/nmeth.4285 PMC5453245

[46] Pei, J. & Grishin, N. V. PROMALS3D: multiple protein sequence alignment enhanced with evolutionary and three-dimensional structural information. *Methods Mol. Biol.* **1079**, 263–271 (2014). 24170408 10.1007/978-1-62703-646-7_17 PMC4506754

