# Supplementary material for: Double-stranded RNA sequencing reveals distinct riboviruses associated with thermoacidophilic bacteria from hot springs in Japan
Source: Nat Microbiol. 2024 Jan 17;9(2):514–23. doi: 10.1038/s41564-023-01579-5 (PMC10847044; doi:10.1038/s41564-023-01579-5)
Supplement: Supplementary file 1 — Supplementary Text, Fig. 1 and Tables 1–3. [file 41564_2023_1579_MOESM1_ESM.pdf]

nature microbiology

Article

https://doi.org/10.1038/s41564-023-01579-5

Double-stranded RNA sequencing reveals distinct riboviruses associated with thermoacidophilic bacteria from hot springs in Japan

In the format provided by the authors and unedited

Sampling sites

The sites H4 and H5 in Hayashida hot spring were located at a natural venting site on the slope of the valley line at the southwestern foot of a caldera lake called Onami Pond in the Kirishima Volcanic complex that is one of the most active volcanic sites in Japan. The sites T1-4 in Tearai area and Y66, Y80, and Y86 in Yunoike area were located at fumerole zone at the western foot of the same caldera pond. The site Oi was located at fumerole zone western foot of the Unsen volcano, which is a volcano that erupted in a devastating eruption that lasted from November 1990 to February 1995[1]. The site Ob was in Obama hot spring area, which is located ~10 km apart coastal area from the site Oi (Supplementary Figure 1).

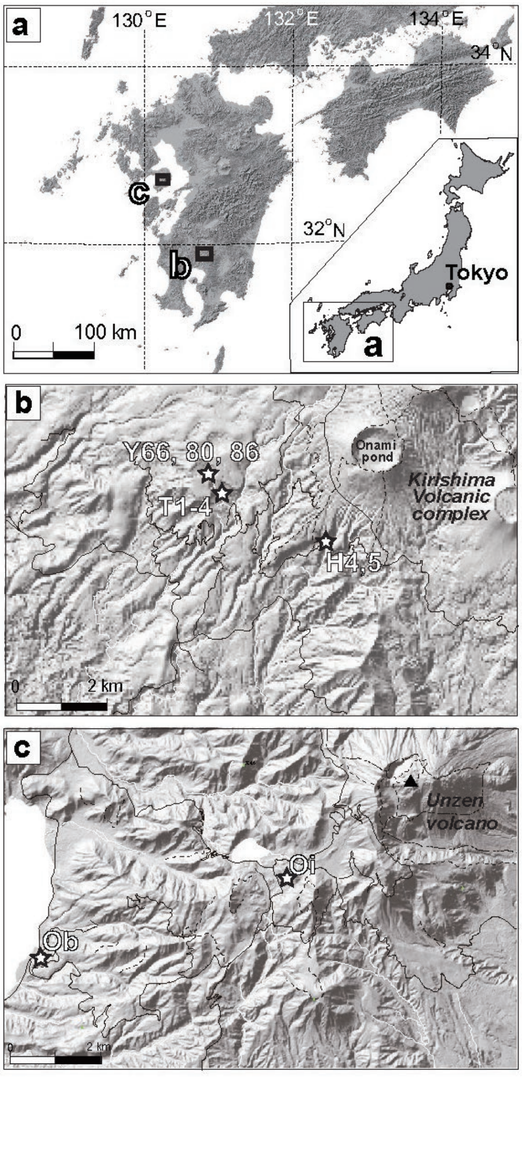

Supplementary Figure 1: Locations of the sampling points. a, Samples were collected from Kyushu, southwestern Japan, where an area with many active volcanoes. **b,** Three sites (H: Hayahida hot spring, T; Tearai area, and Y; Yunoike area), were located on the west side of the Kirishima Volcanic complex. **c,** The sites Oi was located at a fumerole zone western foot of the Unsen volcano. The site Ob differs from the other sites in that it was located along the coast, relatively far from an active volcano. The maps were based on the digital elevation topographic map published by Geospatial Information Authority of Japan（http://maps.gsi.go.jp/）.

Chemistry of hot spring water

The chemical composition of hot spring water was measured and shown in Supplementary Table 1. The major cation (Ca^{2+}, Mg^{2+}, Na^{+}, K^{+}) and anion (Cl^{-}, SO_4^{2-}) were analyzed using ion-chromatography (Gulliver; Jasco International Co., Ltd., Tokyo, Japan) after 21 times dilution of the samples with distilled water. SiO_2 was measured by the molybdenum yellow method with a spectrophotometer. δD and $\delta^{18}O$ were measured from filtered (0.2-μm-pore-size) and non-diluted water samples by using a liquid water isotope analyzer (Los Gatos Research, Inc.). Measurement errors are within 5% for Ca^{2+}, Mg^{2+}, SO_4^{2-}, and SiO_2, 10% for the other ions, ±1.1‰ for δD, and±0.1‰ for $\delta^{18}O$. Table S6 shows the measurement results of the water samples.

Supplementary Table 1: Chemical and isotopic composition of the fluid samples at the 11 samples.

Code	Ca^{2+} (mg/L)	Mg^{2+} (mg/L)	Na^{+} (mg/L)	K^{+} (mg/L)	Cl^{-} (mg/L)	SO_4^{2-} (mg/L)	SiO2 (mg/L)	δD-H_2O (‰ SMOW)	$\delta^{18}O$-H_2O (‰ SMOW)
H4	181	7	77	112	15	33	142	-49.2	-7.1
H5	195	7	80	108	15	33	141	-48.1	-6.9
T1	238	8	995	106	419	111	150	-35.2	-3.3
T2	401	16	81	132	10	602	271	-25.1	-1.5
T3	265	10	77	140	10	170	188	-31.0	-3.0
T4	223	10	62	128	10	304	183	-33.5	-0.7
Y66	195	8	53	117	10	31	92	-40.6	-4.7
Y80	202	8	53	118	10	45	98	-18.8	3.8
Y86	185	8	49	97	30	114	187	-41.9	-4.3
Oi	26	4	22	9	1	431	119	-30.6	-4.4
Ob	113	139	2739	310	1671	131	133	-31.8	-3.8

Composition of SSU rRNA and identification of RNA virus

Eukaryotic SSU rRNA reads represented less than 10% of the microbial communities in all samples. The microbial communities of T1, T2, T4 and Y86 samples, collected from the hot springs with temperatures above 85°C and pH<3, were

predominated by archaea (>80% of total SSU rRNA reads), with thermoacidophilic archaeal family *Sulfolobaceae* representing >50% of the total SSU rRNA reads. By contrast, sequences related to thermoacidophilic bacterial family *Hydrogenobaculaceae* predominated (>50% of total SSU rRNA reads) in samples H4, H5, Y66 and Y80. The microbial community structures in the remaining three stations (T3, Oi and Ob) were distinct from those in the other samples. In particular, betaproteobacterial family *Comamonadaceae* occupied 25% of total SSU rRNA reads in Oi sample, which were negligible in other samples (Extended Data Table 1).

RNA2 and RNA2* of HsRV

Notably, the 5'-terminal three genes in RNA2 and RNA2* encode homologous proteins containing predicted transmembrane domains (BLASTP, *E*-value < 1e-07), so that RNA2 and RNA2* most likely represent two distinct variants of the same segment (Fig. 2a; Extended Data Table 3), with the viruses from the Oi sample having bisegmented genomes. However, we cannot rule out the possibility that RNA2* represents a satellite genome that depends on the bisegmented virus. Three predicted transmembrane proteins that were shared between RNA2 and RNA2* (Fig. 2a) could be involved in virion morphogenesis and/or virus-host interactions[2].

The amino acid sequence of RNA1_ORF4 of HsRV

HMMscan[3] software of HMMER package using Neo-HMM (score >20)[4] and RVDB-HMM (score >20)[5], and palmscan[6] did not identify RNA1_ORF4 as an RdRP. Furthermore, no sequence related to RNA1_ORF4 could be identified using PZLAST[7] among several tera-bytes of public metagenomic sequences either. Similarly, direct comparison of RNA1_ORF4 against the RdRPs from the recently described putative divergent phyla of riboviruses[8-10] revealed no significant similarity (BLASTP, *E*-value ≤ 1e-05). Attempts to generate a structural model of RNA1_ORF4 using AlphaFold2 with a default multiple sequence alignment (MSA) generation method did not result in a reliable model, likely due to the lack of identifiable homologs in public databases.

Proteins of HsRV-like viruses

Similar to HsRV, the RdRPs of these related viruses from moderate aquatic environments did not show similarity to sequences in current databases either using

BLASTP or HHpred searches. However, the functions of several other proteins could be predicted from HHpred search results (Extended Data Fig. 1b). In particular, ORF1 and ORF3 of Ga0393213 encode putative carbohydrate-binding protein with the jelly-roll fold and a phospholipase A2 (PLA2), respectively. Notably, PLA2 has been previously identified in viruses with small RNA and ssDNA genomes[11,12] and, in parvoviruses, shown to be important for infectivity[12]. Ga0169446 encodes a predicted kinase related to nucleoside monophosphate kinases. Finally, Ga0169446 and Ga0456180 encode non-orthologous zinc finger proteins, which could be involved in nucleic acid binding or protein-protein interactions.

Apart from the RdRP, the HsRV-like viruses lack predicted proteins shared with HsRVs from hot springs. However, the three viruses from the moderate environments share a ~500 aa-long protein encoded upstream of the RdRP, a likely candidate for the capsid protein (Fig. 3a). In addition, Ga0456180 and Ga0393213 share a protein of unknown function.

RNA2 of HsPV and its related viruses

ORF1 and ORF2 of RNA2 did not show significant sequence similarity to proteins in sequence or profile databases, except that ORF2 was predicted to encode a membrane protein with two transmembrane domains. Given the relatively high similarity between the RdRP of HsPV and Driatsky virus (Accession: MT025082), we explored the possibility that the second genomic segment of Driatsky virus was missed, not an uncommon situation with segmented RNA viruses, especially for segments not encoding the RdRP. Driatsky virus was identified in a metatranscriptome of an environmental animal sample, but labeled to be "unlikely vertebrate associated". To identify the potential RNA2 of Driatsky virus, we searched contig sequences from the corresponding metatranscriptome (SRR7239362) using HsPV RNA2 as a query. BLASTX analysis against ORF1 of RNA2 identified only one significantly similar sequence, contig_9778 (see below). This contig encompassed two ORFs, as in the case of HsPV RNA2, and the amino acid sequences of contig_9778 ORF1 showed significant similarity (*E*-value = 2e-06) with HsPV-H4 RNA2_c_ORF1 protein.

Unlike in other classified members of the family, RNA2 of HsPV is bicistronic and encodes a membrane protein of unknown function, which is likely to participate in virion morphogenesis and/or virus-host interaction. A similar genome organization has been

observed in the unclassified genPartiti.0019 partiti-like viruses[9]; however, the latter clade is nested among bona fide partitiviruses, forming a sister group to deltapartitiviruses (Fig. 5c).

Contig sequence

>SRR7239362_trimmed_contig_9778 Average coverage: 49.10

GTTCTGTCTAAACACTAGCCTTCCACTTAGTGGTGATAGCTTAATTAGTTAGGTAAACTA
AAGCTGACCGTCCTGTCTCTGGGCCCGCTGGTTGAGGTAACACCCGTATTTAGGACAAC
GTATGTCTGGCCTTCGGGCAAGTCGGCATTCCGCCGGCGATTTCTCGAATCATGGACGA
ATTCGAGTCATTCCGTCAGTCCCGTCCTTATAGGCAGCATAGTGCGCCTGAGGTCTCAG
TACGTAAACTGTTTACTTTGGAGTCTTTATGATTAAACATAATACTCAAGCTGTCAGTGA
TGATAGCATTGAACCTGTTGCCCGTCAGTACGGTCTTCAACTGATGGCACAGGGGGTGG
CAACACTCTCCCAATGTGACGTCTTCGTTCGCCCCATCCCTGGTATCCAATGTGTTGTTG
GTGCTTGGGCTACTCTTACGAAGTTCAAACGAGCTGCTCAAGTTGTTTCCGAGGAACGC
CTCAAAGAACACTTGAACATCGTCACTGCTTTACGCGTCCTTCAAGTTCGTGGTGAGAT
TGATGACCCGCGTATGGATGTTCGTCGCTGTGTCTATCCCTCGATCCTCCGCCCGGTGTT
CCGTGCGATTGGTGATGTGATGGATGAGAGCGTGAGCCTCGATCTTCGCGTTAATCTTT
CCGATGAGCTTGTGGAAGTCATTAAAGGGTATGACTTCGCAAACTGGCGTGACGACAA
CCAAATGATTCAGATCGCTTTAATGCAATCTGGTATCTCCTGCGCTACCTGCCTACCGCC
TGAGGTCGACGGGAATCGTCAGGTGTTGACCATGATCGTTAAGGAGGTCGAGGGTCAG
GCCCGAGCCGAGGGTCAGGTCGTCGGTTTTGATCGTTCAGCGAATCCTGGTGAGGTTCT
GGTCGGAGCCATTTTGGGCCACCGTTTAGAAGATCCTTCCATCTTCGGTGCTCCGCGTGT
CTCATACCAAAACGTCGGGTACTTCGAAGACCAGTTCTATAACTTGGTTAACGCTGAAG
TTCTCAACGCCCGTTCTAAGTAGTGATGAAAGAGAGGTCCAGCGATGAATCTATCCTTA
TTCTTTGTAGCATTTGTAATCTTCTGGTTACTATCTGTGGTCATATGCTTCATGGTAATTG
GGTTCGTTAATGGCCTCTCGTTCGGCTTCGCTTTCCGTTACCTGACCACAGGTGATGGCG
CGTTGCTCGTCACCCTTACAGCCAGTGTATGTGAGATATTCTACTTACTATTCTGGCTTC
ACTGCGTGCTAGATCAGCGTTTGGCAGTCGAGGATGTTTCTCGTCAAGTCGAGAAGATA
CGCACGAGTATCAACAAGATTAGTCGAGACGATTTGTCAAAGCTCAATGCCGTTGTGGA
TGGCGTTAATTCGCTTAATACTGAGGTCCTTCGTCTGGAGAAGGGGCTTGATGAAGCCT
GTGATGTTATGGAAAGGAACATCCCTAGGTTAAGTCAGTCTGCTGACGAGATCGTTCGG
TTCCACAGTGCATTGGACTCCCATGGTGAGGAGTTGAGAGAGATGTTCGTGAATACACT
CGCATCTCGATATCACGAGGTCCTGACGGAAACCTTCAGTGAGACCAAACTAGCACAG

ATACGTGGGTTTGTATCAAGGCAGTCGTTTAAGATTCTCACGTTGCATTTGCCATTCTGT
GAGTATTTCGACTTGTTCACTCCCGAGCAGAAGAAGTTGTACTTCGACTGTATGCGTAG
CGGCGATTACAGACGCCTGTCGAAGAGCCTGACGAAGGAACAGAGAGACGCTGTAAGT
AAGGGTTAGGTCTCCCTTGCGGAGGGGGGG

Metagenomic sequencing and data processing

Five metagenomic libraries were constructed and analyzed as described before[13] (Supplementary Table 2). In brief, DNA was extracted from cells collected on a portion of the 0.2-μm-pore-size filters corresponding to approximately 0.25–2.5 L of H4, H5 and Oi samples, that potential complete RNA virus genomes were identified, using DNeasy PowerSoil Kit (QIAGEN). Covaris M220 (Woburn, MA, USA) was used for physical DNA fragmentation using the conditions described below to obtain a peak fragment size: 400 bp; Peak Power: 75.0, Duty Factor: 15.0, Cycles/Burst: 200, and Time: 60 s. Then, shotgun metagenomic libraries were constructed using KAPA Hyper Prep Kit. The metagenomic sequence libraries were analyzed using the Illumina MiSeq platform with a 2×300-bp read length.

Sequence reads were quality filtered using Trimmomatic v0.35 with the option "LEADING:20 TRAILING:20 MINLEN:60". The quality-controlled reads were then assembled in a sample-by-sample manner using MEGAHIT v1.1.4 with the default setting. Only the long contigs (≥1kb) were retained for further analyses. CRISPR regions were identified using MinCED v0.4.2 with the default setting. The identified CRISPR spacers (n=919) were assembled as a database for the identification of potential virus-host interactions. The 25 virus genome segments were used as a query for sequence similarity search against the CRISPR spacer database. The similarity search was performed using BLASTn with the options "-word_size 7 -evalue 1e-3 -dbsize 100000000" and no sequence met the threshold.

Supplementary Table 2: Hot spring metagenomes and CRISPRs

sample	read bp	assembly size (≥1kb)	number of contigs	CRISPR region	CRISPR spacer
H4	631873847	17009520	8982	19	66
H5	555047200	4338329	1735	15	100
Oi	694063525	16906462	11827	62	207
Y66	622940918	18786560	11513	56	347

GenBank accession numbers of the RdRP proteins from representative members of the family *Partitiviridae* and related sequences

GenBank accession numbers of the RdRP proteins from representative members of the family *Partitiviridae* and related sequences, are shown in Supplementary Table 3.

Supplementary Table 3: Sequences used in phylogenetic analysis of HsPV and related viruses.

Name	Accession	Category
White clover cryptic virus 1	AAU14888.1	Alphapartitivirus
Vicia cryptic virus	AAX39023.1	Alphapartitivirus
Carrot cryptic virus	ACL93278.1	Alphapartitivirus
Heterobasidion partitivirus 3	ACO37245.1	Alphapartitivirus
Heterobasidion partitivirus 1	ADV15441.1	Alphapartitivirus
Heterobasidion partitivirus 12	AHL25151.1	Alphapartitivirus
Heterobasidion partitivirus 13	AHL25153.1	Alphapartitivirus
Heterobasidion partitivirus 15	AHL25162.1	Alphapartitivirus
Helicobasidium mompa dsRNA mycovirus	BAC23065.1	Alphapartitivirus
Helicobasidium mompa partitivirus V1-1	BAD32677.1	Alphapartitivirus
Flammulina velutipes browning virus	BAH56481.1	Alphapartitivirus
Rosellinia necatrix partitivirus 2	BAM78602.1	Alphapartitivirus
Amasya cherry disease-associated mycovirus	CAG77604.1	Alphapartitivirus
Chondrostereum purpureum cryptic virus 1	CAQ53729.1	Alphapartitivirus
Beet cryptic virus 1	YP_002308574.1	Alphapartitivirus
Fusarium poae virus 1	AAC98734.1	Betapartitivirus
Rhizoctonia solani virus 717	AAF22160.1	Betapartitivirus
Heterobasidion annosum P-type partitivirus	AAL79540.1	Betapartitivirus
Pleurotus ostreatus virus 1	AAT07072.1	Betapartitivirus
Ceratocystis resinifera virus 1	AAU26069.1	Betapartitivirus
Primula malacoides virus China/Mar2007	ABW82141.1	Betapartitivirus
Heterobasidion partitivirus 2	ADL66905.1	Betapartitivirus
Cannabis cryptic virus	AET80948.1	Betapartitivirus
Heterobasidion partitivirus 7	AEX87907.1	Betapartitivirus
Heterobasidion partitivirus 8	AFW17810.1	Betapartitivirus
Red clover cryptic virus 2	AGJ83765.1	Betapartitivirus
Hop trefoil cryptic virus 2	AGJ83767.1	Betapartitivirus
Crimson clover cryptic virus 2	AGJ83769.1	Betapartitivirus
Dill cryptic virus 2	AGJ83771.1	Betapartitivirus
Atkinsonella hypoxylon partitivirus	NP_604475.1	Betapartitivirus

Rosellinia necatrix partitivirus 1-W8	YP_392480.1	Betapartitivirus
Cryptosporidium parvum virus 1	ARS33771.1	Cryspovirus
Beet cryptic virus 3	AAB27624.1	Deltapartitivirus
Beet cryptic virus 2	ADP24757.1	Deltapartitivirus
Pepper cryptic virus 2	AEJ07892.1	Deltapartitivirus
Pepper cryptic virus 1	ASU63844.1	Deltapartitivirus
Pittosporum cryptic virus 1	CEJ95596.2	Deltapartitivirus
Fig cryptic virus	YP_004429258.1	Deltapartitivirus
Discula destructiva virus 2	AAK59379.1	Gammapartitivirus
Gremmeniella abietina RNA virus MS1	AAM12240.1	Gammapartitivirus
Penicillium stoloniferum virus F	AAU95758.1	Gammapartitivirus
Aspergillus ochraceous virus	ABV30675.1	Gammapartitivirus
Ophiostoma partitivirus 1	CAJ31886.1	Gammapartitivirus
Discula destructiva virus 1	NP_116716.1	Gammapartitivirus
Fusarium solani virus 1	NP_624350.1	Gammapartitivirus
Penicillium stoloniferum virus S	YP_052856.2	Gammapartitivirus
Grapevine partitivirus	AFX73023.1	
Ustilaginoidea virens partitivirus	AGO04402.1	
Talaromyces marneffei partitivirus-1	AKF14154.1	
Colletotrichum truncatum partitivirus 1	ALF46547.1	
Wuhan insect virus 24	APG78199.1	
Wuhan insect virus 25	APG78200.1	
Wuhan fly virus 6	APG78203.1	
Shuangao partiti-like virus 1	APG78213.1	
Wuhan insect virus 23	APG78216.1	
Hubei partiti-like virus 33	APG78222.1	
Hubei partiti-like virus 39	APG78233.1	
Hubei partiti-like virus 41	APG78238.1	
Hubei partiti-like virus 46	APG78243.1	
Hubei partiti-like virus 29	APG78244.1	
Hubei diptera virus 19	APG78245.1	
Hubei partiti-like virus 36	APG78249.1	
Hubei partiti-like virus 32	APG78251.1	
Hubei odonate virus 13	APG78254.1	
Hubei partiti-like virus 19	APG78260.1	
Hubei partiti-like virus 38	APG78261.1	
Hubei partiti-like virus 31	APG78277.1	
Hubei partiti-like virus 42	APG78281.1	
Hubei partiti-like virus 53	APG78297.1	
Hubei partiti-like virus 54	APG78298.1	
Hubei partiti-like virus 55	APG78299.1	
Hubei partiti-like virus 40	APG78310.1	
Hubei partiti-like virus 34	APG78322.1	
Hubei partiti-like virus 45	APG78330.1	
Wuhan house centipede virus 8	APG78332.1	
Wenling partiti-like virus 3	APG78345.1	
Hubei partiti-like virus 43	APG78364.1	
Araticum virus	ASV45859.1	
Vespa velutina partiti-like virus 1	ATY36109.1	
Moyuka partiti-like virus	AWV66984.1	
Lysoka partiti-like virus	AWV66985.1	
Lysoka partiti-like virus	AWV66996.1	
Lysoka partiti-like virus	AWV67000.1	
Lysoka partiti-like virus	AWV67005.1	
Lysoka partiti-like virus	AWV67007.1	
Lysoka partiti-like virus	AWV67008.1	
Limbe partiti-like virus	AWV67010.1	

Galbut virus	AWY11084.1
Linepithema humile partiti-like virus 1	AXA52551.1
Diatom colony associated dsRNA virus 14	BAU79511.1
Beauveria bassiana partitivirus 1	CUS18591.1
Jam Partiti-like virus 1	GBH21730.1
St73 Partiti-like virus 2	GBH22439.1
St122 Partiti-like virus 1	GBH22945.1
Whatley partiti-like virus	QIJ70094.1
Driatsky virus	QIS87951.1
Senko virus	QIS88027.1
Retsystermes virus	QQM16309.1
Red panda associated partiti-like virus	UBJ26016.1
Fragaria chiloensis cryptic virus	YP_001274391.1
Rose cryptic virus 1	YP_001686786.1
Botryotinia fuckeliana partitivirus 1	YP_001686789.1
Persimmon cryptic virus	YP_006390091.1
Verticillium dahliae partitivirus 1	YP_009164038.1
Beihai barnacle virus 13	YP_009329869.1
genPartiti.0019_ND_045523__367335_1	Neri et al., 2022 [3]
genPartiti.0019_ND_046274__368147_1	Neri et al., 2022
genPartiti.0019_ND_046287__368160_1	Neri et al., 2022
genPartiti.0019_ND_051306__373610_1	Neri et al., 2022
genPartiti.0019_ND_067862__391490_1	Neri et al., 2022
genPartiti.0019_ND_173238__78398_1	Neri et al., 2022
genPartiti.0019_ND_177088__82645_1	Neri et al., 2022
genPartiti.0019_ND_179726__85563_2	Neri et al., 2022
genPartiti.0019_ND_303809__222682_1	Neri et al., 2022
genPartiti.0029_ND_031488__234967_1	Neri et al., 2022
genPartiti.0029_ND_031532__235457_1	Neri et al., 2022
genPartiti.0029_ND_069575__393354_1	Neri et al., 2022
genPartiti.0029_ND_074702__398904_1	Neri et al., 2022
genPartiti.0029_ND_075049__399277_1	Neri et al., 2022
genPartiti.0029_ND_075356__399609_2	Neri et al., 2022
genPartiti.0029_ND_075743__400019_1	Neri et al., 2022
genPartiti.0029_ND_075753__400030_1	Neri et al., 2022
genPartiti.0029_ND_095676__421842_1	Neri et al., 2022
genPartiti.0029_ND_115564__16904_1	Neri et al., 2022
genPartiti.0029_ND_128858__30001_1	Neri et al., 2022
genPartiti.0029_ND_130782__32096_1	Neri et al., 2022
genPartiti.0029_ND_132399__33824_2	Neri et al., 2022
genPartiti.0029_ND_169428__74186_1	Neri et al., 2022
genPartiti.0029_ND_185609__92070_1	Neri et al., 2022
genPartiti.0029_ND_191455__98530_1	Neri et al., 2022
genPartiti.0029_ND_191488__98566_1	Neri et al., 2022
genPartiti.0029_ND_191745__98849_1	Neri et al., 2022
genPartiti.0029_ND_192023__99158_2	Neri et al., 2022
genPartiti.0029_ND_192039__99175_1	Neri et al., 2022
genPartiti.0029_ND_192508__5877_1	Neri et al., 2022
genPartiti.0029_ND_193094__100344_1	Neri et al., 2022
genPartiti.0029_ND_193588__100889_1	Neri et al., 2022
genPartiti.0029_ND_193591__100893_1	Neri et al., 2022
genPartiti.0029_ND_193594__100896_1	Neri et al., 2022
genPartiti.0029_ND_193695__101008_1	Neri et al., 2022
genPartiti.0029_ND_196151__103717_1	Neri et al., 2022
genPartiti.0029_ND_226864__137675_1	Neri et al., 2022
genPartiti.0029_ND_239806__151888_2	Neri et al., 2022
genPartiti.0029_ND_240342__152484_1	Neri et al., 2022

genPartiti.0029_ND_249293__162377_1	Neri et al., 2022
genPartiti.0029_ND_288844__206125_1	Neri et al., 2022
genPartiti.0029_ND_298891__217236_1	Neri et al., 2022
genPartiti.0029_ND_299662__218087_1	Neri et al., 2022
genPartiti.0029_ND_300928__219487_1	Neri et al., 2022
genPartiti.0029_ND_304500__223450_1	Neri et al., 2022
genPartiti.0029_ND_310346__229934_1	Neri et al., 2022
genPartiti.0029_ND_314423__234461_1	Neri et al., 2022
genPartiti.0029_ND_343623__266851_1	Neri et al., 2022
genPartiti.0029_ND_346095__269594_1	Neri et al., 2022
genPartiti.0029_ND_346121__269624_1	Neri et al., 2022
genPartiti.0029_ND_346159__269665_1	Neri et al., 2022
genPartiti.0029_ND_349204__16683_1	Neri et al., 2022
genPartiti.0029_ND_357189__281891_1	Neri et al., 2022
HsPV-H4_RNA1_a_ORF1	This study.
HsPV-H4_RNA1_b_ORF1	This study.
HsPV-H5_RNA1_a_ORF1	This study.
HsPV-Y66_RNA1_a_ORF1	This study.
HsPV-Y66_RNA1_b_ORF1	This study.

Discussion

This work continues the trend established by recent large scale metatranscriptome surveys that substantially expanded the prokaryotic RNA virome as well as the ecological range of RNA viruses[8,9]. Unlike most of those large-scale studies, however, here we report complete genomes of multi-segmented riboviruses, adding information on their genome organization and gene content, beyond the RdRP. Some groups of riboviruses, such as picobirnaviruses and certain branches of partitiviruses, previously thought to infect eukaryotes, have been reassigned to prokaryotic hosts through a combination of evidence including their recovery from habitats that are heavily dominated by prokaryotes, genome organization, the presence of SD motifs, and in some cases, encoding of cell wall lysis enzymes and CRISPR targeting[9]. Here we describe complete genomes of partiti-like viruses from the expansive genPartiti.0029 group that apparently infect a bacterial host, most likely, the extremely thermophilic *Hydrogenobaculum* sp. Previously, genPartiti.0019 viruses, which shows a specific evolutionary affinity with Deltapartitiviruses that have well established eukaryotic hosts, were predicted to infect moderately thermophilic bacteria of the genus *Roseiflexus* based on the CRISPR matches[9]. Thus, our findings support the previous conclusions on multiple switches from eukaryotic to prokaryotic hosts (and vice versa) among partiti-like viruses and further suggest that these viruses can also switch between mesophilic and thermoacidophilic hosts.

Apart from the unusual RdRP, the distinctness of this group is emphasized by the lack of functional prediction for the rest of their proteins. In particular, there is no clarity

as to the identity of their capsid proteins and accordingly, the virion structure that remains to be studied experimentally. We note, however, that ORF3 of RNA1, which encodes the largest protein of the virus, is a likely candidate for one of the major capsid proteins. Similar to the inner capsid protein P1 of cystoviruses[14], the protein is encoded on the same segment (RNA1) as the RdRP. The other conserved non-membrane proteins encoded on segment RNA2 (and RNA2*) could constitute additional virion components. Similarly, the HsRV-like viruses from moderate ecosystems also encode a conserved protein at the equivalent position, which could represent their capsid protein. Although the two groups of potential capsid proteins do not share appreciable sequence similarity and substantially differ in length (~1000 aa vs ~500 aa), we cannot exclude the possibility that they are extremely divergent homologs.

The discovery of RNA viruses in acidic and high temperature conditions seems unexpected given the fragility of long RNA molecules. However, genome segmentation, with relatively small individual segments, and the likely double-stranded genome structure, which is associated with higher thermal stability[15], apparently obviate this problem. Given these findings, it becomes particularly puzzling why no RNA viruses have been so far linked to archaea. A ribovirus genome has been previously sequenced from a Yellowstone hot spring and tentatively assigned a hyperthermophilic archaeal host[16]. Subsequently, however, numerous related ribovirus genomes have been identified in a mesophilic environment[11], whereas there was no follow-up on putative archaeal virus; thus, the status of this finding remains uncertain. One possibility is that such viruses remain unrecognized because they encode extremely diverged RdRPs or even distinct RdRPs unrelated to those encoded by the members of the *Riboviria*.

Reference

1 Nakada, S., Shimizu, H. & Ohta, K. Overview of the 1990–1995 eruption at Unzen Volcano. *Journal of Volcanology and Geothermal Research* **89**, 1–22 (1999).

2 Bamford, D. H., Romantschuk, M. & Somerharju, P. Membrane fusion in prokaryotes: bacteriophage phi 6 membrane fuses with the Pseudomonas syringae outer membrane. *The EMBO journal* **6**, 1467-1473 (1987).

3 Johnson, L. S., Eddy, S. R. & Portugaly, E. Hidden Markov model speed heuristic and iterative HMM search procedure. *BMC Bioinformatics* **11**, 1-8 (2010).

4 Sakaguchi, S. *et al.* NeoRdRp: A Comprehensive Dataset for Identifying RNA-dependent RNA Polymerases of Various RNA Viruses from Metatranscriptomic Data. *Microbes Environ.* **37**, doi:10.1264/jsme2.ME22001 (2022).

5 Bigot, T., Temmam, S., Pérot, P. & Eloit, M. RVDB-prot, a reference viral protein database and its HMM profiles. *F1000Res* **8**, 530, doi:10.12688/f1000research.18776.2 (2019).

6 Babaian, A. & Edgar, R. Ribovirus classification by a polymerase barcode sequence. *PeerJ* **10**, e14055 (2022).

7 Mori, H. *et al.* PZLAST: an ultra-fast amino acid sequence similarity search server against public metagenomes. *Bioinformatics* **37**, 3944-3946 (2021).

8 Zayed, A. A. *et al.* Cryptic and abundant marine viruses at the evolutionary origins of Earth's RNA virome. *Science* **376**, 156-162, doi:10.1126/science.abm5847 (2022).

9 Neri, U. *et al.* Expansion of the global RNA virome reveals diverse clades of bacteriophages. *Cell* **185**, 4023-4037 (2022).

10 Forgia, M. *et al.* Hybrids of RNA viruses and viroid-like elements replicate in fungi. *Nat Commun* **14**, 2591, doi:10.1038/s41467-023-38301-2 (2023).

11 Wolf, Y. I. *et al.* Doubling of the known set of RNA viruses by metagenomic analysis of an aquatic virome. *Nat Microbiol* **5**, 1262-1270, doi:10.1038/s41564-020-0755-4 (2020).

12 Zádori, Z. *et al.* A viral phospholipase A2 is required for parvovirus infectivity. *Dev. Cell* **1**, 291-302 (2001).

13 Hirai, M. *et al.* Library Construction from Subnanogram DNA for Pelagic Sea Water and Deep-Sea Sediments. *Microbes Environ.* **32**, 336-343, doi:10.1264/jsme2.ME17132 (2017).

14 Poranen, M. M., Mantynen, S. & Ictv Report, C. ICTV Virus Taxonomy Profile: Cystoviridae. *J. Gen. Virol.* **98**, 2423-2424, doi:10.1099/jgv.0.000928 (2017).

15 Dubins, D. N., Lee, A., Macgregor, R. B. & Chalikian, T. V. On the stability of double stranded nucleic acids. *J. Am. Chem. Soc.* **123**, 9254–9259 (2001).

16 Bolduc, B. *et al.* Identification of novel positive-strand RNA viruses by metagenomic analysis of archaea-dominated Yellowstone hot springs. *J. Virol.* **86**, 5562–5573, doi:10.1128/JVI.07196-11 (2012).
