# Supplementary material for: Double-stranded RNA sequencing reveals distinct riboviruses associated with thermoacidophilic bacteria from hot springs in Japan
Source: Nat Microbiol. 2024 Jan 17;9(2):514–23. doi: 10.1038/s41564-023-01579-5 (PMC10847044; doi:10.1038/s41564-023-01579-5)
Supplement: Supplementary file 2 — Reporting Summary [file 41564_2023_1579_MOESM2_ESM.pdf]

nature portfolio

Corresponding author(s): Syun-ichi Urayama

Last updated by author(s): Nov 27, 2023

Reporting Summary

Nature Portfolio wishes to improve the reproducibility of the work that we publish. This form provides structure for consistency and transparency in reporting. For further information on Nature Portfolio policies, see our Editorial Policies and the Editorial Policy Checklist.

Statistics

For all statistical analyses, confirm that the following items are present in the figure legend, table legend, main text, or Methods section.

n/a	Confirmed	
☒	☐	The exact sample size (*n*) for each experimental group/condition, given as a discrete number and unit of measurement
☐	☒	A statement on whether measurements were taken from distinct samples or whether the same sample was measured repeatedly
☒	☐	The statistical test(s) used AND whether they are one- or two-sided<br>*Only common tests should be described solely by name; describe more complex techniques in the Methods section.*
☒	☐	A description of all covariates tested
☒	☐	A description of any assumptions or corrections, such as tests of normality and adjustment for multiple comparisons
☒	☐	A full description of the statistical parameters including central tendency (e.g. means) or other basic estimates (e.g. regression coefficient) AND variation (e.g. standard deviation) or associated estimates of uncertainty (e.g. confidence intervals)
☒	☐	For null hypothesis testing, the test statistic (e.g. *F*, *t*, *r*) with confidence intervals, effect sizes, degrees of freedom and *P* value noted<br>*Give P values as exact values whenever suitable.*
☒	☐	For Bayesian analysis, information on the choice of priors and Markov chain Monte Carlo settings
☒	☐	For hierarchical and complex designs, identification of the appropriate level for tests and full reporting of outcomes
☒	☐	Estimates of effect sizes (e.g. Cohen's *d*, Pearson's *r*), indicating how they were calculated

Our web collection on statistics for biologists contains articles on many of the points above.

Software and code

Policy information about availability of computer code

Data collection	No software was used for data collection.
Data analysis	A custom Perl pipeline script used for read clenup is available at GitHub (https://github.com/takakiy/FLDS).<br><br>The following commercial programs were used.<br>CLC GENOMICS WORKBENCH version 11.0 (Qiagen Japan, Tokyo, Japan); Genetyx version 14 (Genetyx, Tokyo, Japan)<br><br>The following open source programs were used.<br>Tablet viewer (version 1.19.09.03); phyloFlash (version 3.4); BLASTX (version 2.2.31+); Prodigal (version 2.6.3); HHpred (online server [no versions]); MEGA6.06; TMHMM (version 2.0); ColabFold 1.5.1; AlphaFold 2 through ColbFold v1.5.2; DALI (online, DaliLite.v5); ChimeraX (version 1.5); trimAl (version 1.4.rev15); RAxML (8.2.10); ProtTest (version 3.4.2); PROMALS3D; IQ-TREE (version 2.0.6); MAFFT (version 7); BLASTP (v2.9.0); HHblits (v3.3.0); ModelFinder (a part of IQ-TREE); BLASTn/p/x (2.12.0+)

For manuscripts utilizing custom algorithms or software that are central to the research but not yet described in published literature, software must be made available to editors and reviewers. We strongly encourage code deposition in a community repository (e.g. GitHub). See the Nature Portfolio guidelines for submitting code & software for further information.

Data

Policy information about availability of data

All manuscripts must include a data availability statement. This statement should provide the following information, where applicable:

- Accession codes, unique identifiers, or web links for publicly available datasets
- A description of any restrictions on data availability
- For clinical datasets or third party data, please ensure that the statement adheres to our policy

Datasets obtained in this study have been available in the GenBank database repository (Accession Nos. HsRV: BTCN01000001-BTCN01000010; HsPV-H4: BTCO01000001-BTCO01000006; HsPV-H5: BTCP01000001-BTCP01000005; HsPV-Y66:BTCQ01000001-BTCQ01000004; H5_contig_1: BTCR01000001; Oi_contig_1-9: BTCS01000001-BTCS01000009) and Short Read Archive database (Accession No. DRA016131). Datasets (PDB70 [mmcif_2023-10-24], Pfam [v35], UniProt-SwissProt-viral70_Nov_2021 and NCBI-CD [v3.19]) are available at http://ftp.tuebingen.mpg.de/pub/protevo/toolkit/databases/hhsuite_dbs/. Searches using the IMG/VR dataset were available only at https://img.jgi.doe.gov/cgi-bin/vr/main.cgi?section=WorkspaceBlast&page=viralform. Datasets (SILVA SSU [version 138], Neo-HMM [v1.1], and RVDB-HMM [v23.0]) are publicly available.

Research involving human participants, their data, or biological material

Policy information about studies with human participants or human data. See also policy information about sex, gender (identity/presentation), and sexual orientation and race, ethnicity and racism.

Reporting on sex and gender	This research does not involve human participants, their data, or biological material.
Reporting on race, ethnicity, or other socially relevant groupings	This research does not involve human participants, their data, or biological material.
Population characteristics	This research does not involve human participants, their data, or biological material.
Recruitment	This research does not involve human participants, their data, or biological material.
Ethics oversight	This research does not involve human participants, their data, or biological material.

Note that full information on the approval of the study protocol must also be provided in the manuscript.

Field-specific reporting

Please select the one below that is the best fit for your research. If you are not sure, read the appropriate sections before making your selection.

☐ Life sciences ☐ Behavioural & social sciences ☒ Ecological, evolutionary & environmental sciences

For a reference copy of the document with all sections, see nature.com/documents/nr-reporting-summary-flat.pdf

Ecological, evolutionary & environmental sciences study design

All studies must disclose on these points even when the disclosure is negative.

Study description	This study collected microbes in hot spring water and performed sequencing analyses for RNA virus discovery.
Research sample	Microbes in hot spring water.
Sampling strategy	No sample-size calculations were performed.
Data collection	The chemical composition of hot spring water was measured by T.O. Sequencing data were obtained using Illumina Miseq platform by M.H.
Timing and spatial scale	Sample were collected at 09- or 10-Mar-2017 and 17- or 18-Nov-2015. Each sample was collected once.
Data exclusions	Data from two sampling points were not included in analyses since we could not obtain data from these two samples.
Reproducibility	For data analyses, all raw data is available in the GenBank database repository. Reproducibility of environmental samples and sequencing was not confirmed.
Randomization	No randomization was performed and no controlling for covariants is relevant to this study design.
Blinding	Blinding does not apply to this study since it is discovery-oriented.

Did the study involve field work? ☒ Yes ☐ No

Field work, collection and transport

Field conditions	The weather was sunny or cloudy.
Location	Locations of the samplings are follow;<br>H4: 31°54'07.5"N 130°50'06.2"E<br>H5: 31°54'07.5"N 130°50'06.2"E<br>T1-4: 31°54'37.7"N 130°49'00.6"E<br>Y66, Y80, Y86: 31°55'03.8"N 130°48'40.4"E<br>Oi: 32°44'25.3"N 130°15'48.4"E<br>Ob: 32°43'33.0"N 130°12'24.7"E
Access & import/export	All samples were obtained with the permission of the landowner (or official manager) and in compliance with national law. The issuer are as follow; Unzen City, Unzen Nature Conservation Bureau, Kirishima Iwasaki Hotel, NIPPON PAPER LUMBER CO. LTD. and NITTETSU MINING CO. LTD KAGOSHIMA GEOTHERMAL FACILITY.
Disturbance	Sampling was done with a minimal number of people and collected from ample spring water sources.

Reporting for specific materials, systems and methods

We require information from authors about some types of materials, experimental systems and methods used in many studies. Here, indicate whether each material, system or method listed is relevant to your study. If you are not sure if a list item applies to your research, read the appropriate section before selecting a response.

Materials & experimental systems

n/a	Involved in the study	
☒	☐	Antibodies
☒	☐	Eukaryotic cell lines
☒	☐	Palaeontology and archaeology
☐	☒	Animals and other organisms
☒	☐	Clinical data
☒	☐	Dual use research of concern
☒	☐	Plants

Methods

n/a	Involved in the study	
☒	☐	ChIP-seq
☒	☐	Flow cytometry
☒	☐	MRI-based neuroimaging

Animals and other research organisms

Policy information about studies involving animals; ARRIVE guidelines recommended for reporting animal research, and Sex and Gender in Research

Laboratory animals	This study did not involve laboratory animals.
Wild animals	This study did not involve wild animals.
Reporting on sex	This study did not involve sex information.
Field-collected samples	A total of 11 samples were collected from five hot springs regions at southern Japan, in close proximity to active volcanoes, according to the instructions of Unzen City, Unzen Nature Conservation Bureau and private companies that maintain each hot spring region. At each sampling station, approximately 10 L of hot spring water was collected in a sterilized plastic bag, and then filtered with 0.2-µm-pore-size cellulose acetate membrane filters in 47 mm diameter (Advantec, Tokyo, Japan) within 0.5-3 hours after sampling. The filters were stored at −80°C until nucleic acid extraction.
Ethics oversight	No ethical approval or guidance was required

Note that full information on the approval of the study protocol must also be provided in the manuscript.
